# Cost-effectiveness of tolvaptan for the treatment of hyponatraemia secondary to syndrome of inappropriate antidiuretic hormone secretion in Sweden

**DOI:** 10.1186/s12902-016-0104-z

**Published:** 2016-05-16

**Authors:** Clare Jamookeeah, Paul Robinson, Karl O’Reilly, Johan Lundberg, Martin Gisby, Michael Ländin, Jakob Skov, David Trueman

**Affiliations:** Abacus International, Bicester, Oxfordshire UK; Otsuka Pharmaceutical Europe Ltd., Gallions, Wexham Springs, Framewood Road, Wexham, SL3 6PJ UK; Otsuka Pharma Scandinavia AB, Stockholm, Sweden; Department of Medicine, Karlstad Central Hospital, Karlstad, Sweden

**Keywords:** Hyponatraemia, Tolvaptan, Cost-effectiveness, Cost-utility, Discrete event simulation, SIADH

## Abstract

**Background:**

Tolvaptan is the only vasopressin V_2_ receptor antagonist licensed by the European Medicines Agency for the treatment of hyponatraemia (HN) secondary to the syndrome of inappropriate antidiuretic hormone secretion (SIADH). We have investigated the cost-effectiveness of tolvaptan versus no active treatment (NAT) in adult patients within the licensed indication who have either failed to respond to fluid restriction or for whom the use of fluid restriction is not suitable, from the societal perspective in Sweden.

**Methods:**

A cost-utility analysis, considering a ‘general SIADH’ population and two subpopulations of patients (small-cell lung cancer [SCLC] and pneumonia) to broadly represent the complex clinical pathway of SIADH, was performed. A discrete event simulation was developed to model the progression of individuals through inpatient admissions over a 30-day time horizon (180 days for the SCLC cohort). Clinical data were derived from tolvaptan trials and observational data sources. All costs are given in Swedish kronor (SEK).

**Results:**

In the ‘general SIADH’ population, tolvaptan was associated with reduced costs (SEK 5,779 per patient [€624]) and increased quality-adjusted life-years (QALYs) (0.0019) compared with NAT and was therefore the dominant treatment strategy. Tolvaptan was also associated with reduced costs and increased QALYs in the SCLC and pneumonia subpopulations. The most influential variables in our analysis were reduction in hospital length of stay, duration of treatment and long term treatment with tolvaptan in SCLC patients.

**Conclusions:**

Tolvaptan represents a cost-effective treatment option in Sweden for hospitalised patients with HN secondary to SIADH who have either failed to respond to or are unsuitable for fluid restriction.

**Electronic supplementary material:**

The online version of this article (doi:10.1186/s12902-016-0104-z) contains supplementary material, which is available to authorized users.

## Background

Hyponatraemia (HN) is the most common electrolyte disturbance in clinical practice, occurring in approximately 15–30 % of hospitalised patients [1, 2]. It is commonly defined as a serum sodium concentration ([Na^+^]) <135 mmol/L [2–5], with severe HN defined as <125 mmol/L [2]. Hyponatraemia is associated with increased mortality, morbidity and length of hospital stay [2].

Hyponatraemia can be euvolaemic (i.e. without an associated change in body fluid volume), hypervolaemic (i.e. body fluid volume is increased), or hypovolaemic (body fluid volume is decreased). These three types of HN have different sets of possible aetiologies. The most common cause of euvolaemic HN is the syndrome of inappropriate antidiuretic hormone secretion (SIADH) [3, 6], which is the focus of our analysis. In SIADH water retention is caused by inappropriate increased release of, or responsiveness to, antidiuretic hormone (ADH) [7]. This can be due to malignancy, pneumonia, central nervous system disorders or certain drugs [7]. Although SIADH can be associated with many different types of malignancy, it is most commonly observed with small cell lung cancer (SCLC) and studies have shown a higher mortality rate in SCLC patients with HN than in those without [8, 9]. Drug-induced SIADH usually resolves upon discontinuation of the medication [7].

Swedish guidelines for HN, published by the Swedish Endocrine Society and the Swedish Association for Anaesthesiology and Intensive Care [1], recommend resolution of the underlying condition as the primary treatment strategy in patients with SIADH. If symptoms are mild, HN secondary to SIADH may be treated with fluid restriction. Diagnosis and treatment should be re-evaluated if the patient does not respond within 12–24 h. If the symptoms are more pronounced the patient will require treatment in intensive care [1]. In patients who do not respond to or cannot be treated with fluid restriction, symptoms may exacerbate and patient outcomes may be signficiantly affected. For patients in whom fluid restriction has been unsuccessful or is unsuitable, vasopressin receptor antagonists (i.e., tolvaptan) are recommended [1]. In clinical practice, subjects with HN, especially if it is associated with no or mild symptoms, or if initial treatment was unsuccessful, may often not receive treatment [10].

Tolvaptan, an oral vasopressin V_2_ receptor antagonist, is the only vaptan approved by the European Medicines Agency (EMA) for the treatment of adult patients with HN secondary to SIADH in 2009 [11]. Tolvaptan blocks binding of ADH to the V_2_ receptor which induces electrolyte-free water excretion. The result is a reduction in body water without loss of body electrolytes, which leads to an increase in serum [Na^+^] [4, 5].

The cost-effectiveness of tolvaptan for the treatment of HN secondary to SIADH has never been studied in a European setting. Worldwide, three studies have previously evaluated the potential cost impact of tolvaptan in HN [12–14]; however, one of these was performed in a different indication (heart failure) (14). Of the remaining two studies, Dasta et al. [13] considered a US hospital population with HN secondary to SIADH and observed a total cost offset, including the acquisition cost of tolvaptan, of $694 per admission. The authors estimated that tolvaptan would be cost-neutral with duration of therapy of ≥6.78 days. In a South Korean analysis, Lee et al. [14] found that, in patients hospitalised for treatment and monitoring of euvolaemic and hypervolaemic HN, tolvaptan treatment was less costly and more efficacious compared with placebo.

We have performed an economic evaluation, from a Swedish societal perspective, to determine the cost-effectiveness of tolvaptan, compared with no active treatment (NAT) for HN secondary to SIADH in adult patients who have either failed to respond to fluid restriction or for whom the use of fluid restriction is not suitable.

## Methods

### Design of the economic evaluation

A cost-utility analysis was conducted. The primary outcome was the incremental cost-effectiveness ratio (ICER) expressed as a cost per quality-adjusted life-year (QALY) in line with Tandvårds- och läkemedelsförmånsverket (The Dental and Pharmaceutical Benefits Agency [TLV]) guidance at the time of analysis [15]. The base case used a societal perspective, also in line with TLV guidance.

Tolvaptan was compared with NAT. This choice was based on advice from clinicians and evidence from the HN registry, a multi-centre, prospective, observational study. The HN registry showed that, of patients provided with fluid restriction as a first therapy, 56 % experienced a total increase of [Na^+^] of ≤5 mEq/L and of these patients, 56 % were not provided a second therapy [10]. This suggests that NAT is a relevant option widely used in clinical practice, despite being inappropriate in many cases.

The economic evaluation considered a hypothetical cohort of individuals with HN secondary to SIADH who had either failed to respond to or were unsuitable for fluid restriction. This population was chosen in line with current Swedish guidance and clinical practice [1, 16]. For the purpose of the economic evaluation, HN is defined as [Na^+^] <135 mmol/L and sodium correction is defined as [Na^+^] ≥135 mmol/L, consistent with the tolvaptan clinical trial programme [4, 5].

Three clinically relevant patient populations were evaluated in the analysis: all patients with HN secondary to SIADH (‘general SIADH’); the subgroup of patients with SCLC and HN secondary to SIADH (SCLC cohort); the subgroup of patients with pneumonia and HN secondary to SIADH (pneumonia cohort). The SCLC and pneumonia populations were selected as examples of two highly relevant subpopulations, for which the clinical and patient pathways are very different. They were chosen to broadly represent the complex clinical pathway of SIADH.

A 30-day time horizon was chosen in the base case analysis for the ‘general SIADH’ population, reflecting the relatively short duration of treatment for HN secondary to SIADH and the duration of the tolvaptan Phase III clinical studies [4, 5]. The 30-day time horizon is also thought to capture all relevant consequences for most patients in this population. Similarly, a 30-day time horizon was also deemed appropriate for the pneumonia subgroup, due to the short duration of treatment for HN secondary to SIADH in this population. However, based on clinical expert opinion, a 180-day time horizon was assumed for the SCLC population.

Future costs and benefits are discounted at a rate of 3.0 % per annum, in line with current TLV guidance, but due to the short time horizon of the analysis this is mainly applicable to scenario analyses [15].

### Outline of the model

An individual-level model (specifically, a discrete event simulation [DES]), which simulates multiple clinical outcomes over time was developed to model the progression of individuals, one at a time, through multiple inpatient admissions over the defined time horizon. The DES approach was adopted in preference to a cohort model (which is more commonly seen in this type of analysis) due to the ability to easily incorporate time dependency. For example, many economic and clinical aspects of HN secondary to SIADH may occur at rates that change over time (time to hospital discharge; time to mortality; time to hospital readmission; time to discontinuation of tolvaptan).

**Fig. 1:**
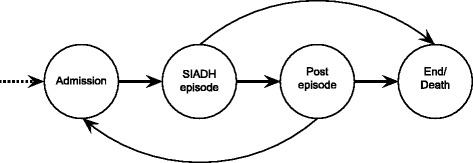
Event graph depicting flow of patients in model

The flow of hypothetical individuals through the model and the order in which events may occur are depicted in Fig. 1. In summary:

1. An individual is admitted to hospital and is diagnosed with HN secondary to SIADH
2. The individual undergoes active treatment or receives NAT for HN secondary to SIADH
3. The individual may undergo further treatment for their underlying condition (such as chemotherapy) before being discharged
  - In the base case, all individuals are assumed to be discharged to home
  - Individuals are assumed to have their underlying condition resolved (‘general SIADH’ and pneumonia) or not (SCLC) at discharge and this affects quality of life (QoL) between admissions
4. The individual may die, either during hospitalisation or following discharge
5. The individual may be readmitted at a later date and will flow through the model again
6. The individual leaves the model at the end of the set time horizon, or at death, whichever occurs first, and a new patient is selected.

The DES was run for 100,000 hypothetical patients to arrive at a set of outputs representative of a cohort in each subpopulation, both with and without tolvaptan treatment. Comparative modelled outputs, other than QALYs, included hospital length of stay (LOS), readmissions, and mortality.

### Data sources

The economic evaluation relies on evidence from several sources, listed below. A summary of how these sources inform the required clinical parameters, across the three base case analyses, is presented in Table 1.

**Table 1:** Application of clinical data sources in the model base cases

	Population modelled		
Parameter	General SIADH	SCLC	Pneumonia
Population characteristics	HN Registry and ART	HN Registry and ART	HN Registry and ART
[Na^+^] correction	HN Registry and SALT I & II	HN Registry and SALT I & II	HN Registry and SALT I & II
Duration of tolvaptan treatment	HN Registry	Expert clinical opinion	Expert clinical opinion
HRQL change	SALT I & II	SALT I & II	SALT I & II
Length of inpatient stay	SALT I & II	SALT I & II	SALT I & II
Inpatient mortality	ART	ART	ART
Readmission	ART	ART	ART
Long-term mortality	Swedish life-tables	ART	Swedish life-tables

*Abbreviations: ART* the Assessment of epidemiology, patient characteristics and outcomes Related To patients with hyponatraemia/SIADH in Sweden, *HN* hyponatraemia, *HRQL* health related quality of life, *SALT* the Study of Ascending Levels of Tolvaptan in Hyponatremia, *SCLC* small cell lung cancer, *SIADH* syndrome of inappropriate antidiuretic hormone secretion

- The Study of Ascending Levels of Tolvaptan in Hyponatremia I and II (SALT I and SALT II) are both randomised, placebo-controlled, double-blind trials examining the effect of tolvaptan on hypervolaemic and euvolaemic HN of different aetiologies [4, 5].
- The Assessment of epidemiology, patient characteristics and outcomes Related To patients with HN/SIADH in Sweden: a population-based register study (ART) is a retrospective cohort study conducted using the compulsory population-based health registers governed by the National Board of Health and Welfare [17].
- The HN Registry is a global, multicentre (US and European), prospective, observational study of current treatment approaches for hospitalised patients with euvolaemic or hypervolaemic HN [10].

### Model inputs

A summary of base case inputs is presented in Table 2. Health-related QoL (HRQL, in the form of health state utilities) for the ‘general SIADH’ population were obtained by mapping SF-12 (12-Item Short Form Health Survey) responses from SALT I and II to EQ-5D (a standardised health outcome questionnaire) [18] using a mapping algorithm developed by Gray et al. [19]. Simulated EQ-5D scores were then used to estimate changes in EQ-5D from baseline at Day 30 using ordinary least squares regression as a function of baseline characteristics, treatment arm and achievement of [Na^+^] correction at Day 4 (≥135 mmol/L) (i.e. the definition of sodium correction, in line with a pre-defined endpoint in SALT I & II [4]). Literature sources were sought to estimate the baseline utility scores for the SCLC and pneumonia populations [20, 21]. Unit costs were collected from a number of different sources including hospital price lists [22], TLV official price database [23] and Statistics Sweden [24]. The reference year of the costs was 2013, and costs are given in Swedish kronor (SEK). Additional details are provided in the Additional file 1.

**Table 2:** Base case inputs by population (data value or type; source)

Model element	General SIADH	SCLC	Pneumonia
Clinical parameters			
Mean age (years)	70; ART (with SIADH)^a^	64; ART (SCLC with SIADH)^a^	69; ART (pneumonia with SIADH)^a^
% Male	33 %; ART (with SIADH)^a^	31 %; ART (SCLC with SIADH)^a^	43 %; ART (pneumonia with SIADH)^a^
Baseline [Na^+^]	125; HN Registry^b^	123; HN Registry [38]	125; HN Registry^b^
Baseline probability [Na^+^] correction (≥135 mmol/L)	Binary logistic regression; HN Registry (SIADH)^b^	Binary logistic regression; HN Registry (SIADH)^b^	Binary logistic regression; HN Registry (SIADH)^b^
Odds ratio normalisation tolvaptan vs. NAT	11.50; SALT I & II [4]	11.50; SALT I & II [4]	11.50; SALT I & II [4]
Duration of tolvaptan treatment	Mean 3 days; Lognormal survival distribution; HN Registry (SIADH)^b^	Mean 4 days; Petereit et al. [28]	Mean 3 days; clinical opinion
Early tolvaptan treatment discontinuation rule	2 days	2 days	2 days
Mean hospital LOS NAT	8.00; SALT I & II (SIADH with [Na^+^] <130 mmol/L) [4]	8.00; SALT I & II (SIADH with [Na^+^] <130 mmol/L) [4]	8.00; SALT I & II (SIADH with [Na^+^] <130 mmol/L) [4]
Percentage reduction hospital LOS with tolvaptan	20.0 %; SALT I & II (SIADH with [Na^+^] <130 mmol/L)^c^	20.0 %; SALT I & II (SIADH with [Na^+^] <130 mmol/L)^c^	20.0 %; SALT I & II (SIADH with [Na^+^] <130 mmol/L)^c^
Probability inpatient mortality	2.2 %; ART (with SIADH)^a^	4.4 %; ART (SCLC with SIADH)^a^	6.2 %; ART (pneumonia with SIADH)^a^
Long-term survival	Based on age; Swedish life-tables [24]	Based on disease; Gompertz distribution from ART (SCLC with SIADH)	Based on age; Swedish life-tables [24]
Time to readmission	Weibull distribution; ART (with SIADH)^a^	Weibull distribution; ART (SCLC with SIADH)^a^	Weibull distribution; ART (pneumonia with SIADH)^a^
Probability of SIADH (and HN) resolution at end of inpatient admission	100 %; assumption	88 %; List et al. [39]	100 %; assumption
Time to resolution^d^	NA in base case	NA in base case	NA in base case
Costs			
Tolvaptan dosing/cost assumption	15/30 mg once daily^e^	15/30 mg once daily^e^	15/30 mg once daily^e^
Care setting for admission	Internal medicine clinic	Oncology clinic	Pulmonary care clinic
Discharge location	Home	Home	Home
Palliative care costs	None	Applied for a maximum of 84 days (3 months)	None
Chemotherapy costs	None	Applied once per inpatient stay	None
Percentage patients in work between readmissions	17 %; [40]	0 %; Assumption	17 %; [40]
HRQL			
Baseline utility	0.58; SALT I & II [41]	0.61; Loveman et al. [20] with HN reduction from SALT I & II [41]	0.73; Schuetz et al. [21] with HN reduction from SALT I & II [41]
Treatment-specific change in EQ-5D at Day 4	Absolute change in simulated EQ-5D at Day 30 from SALT I & II [41]	Absolute change in simulated EQ-5D at Day 30 from SALT I & II [41]	Absolute change in simulated EQ-5D at Day 30 from SALT I & II [41]
Model structure			
Time horizon	30 days	180 days	30 days

*Abbreviations: ART* The Assessment of epidemiology, patient characteristics and outcomes Related To patients with hyponatraemia/SIADH in Sweden: a population-based register study, *HN* hyponatraemia, *HRQL* health-related quality of life, *LOS* length of stay, *NA* not applicable, *NAT* no alternative treatment, *SALT* study of ascending levels of Tolvaptan in Hyponatremia, *SCLC* small cell lung cancer, *SIADH* syndrome of inappropriate antidiuretic hormone secretion

^a^Based on data from ART (unpublished observations)

^b^Analysis of HN registry data

^c^post-hoc analysis of SALT1 and SALT-2

^d^If resolution did not occur as an inpatient

^e^15/30 mg daily associated with equal cost

### Key assumptions

Assumptions had to be made where no suitable data sources could be identified. Key assumptions concerned the benefit of correction of HN, which was conservatively assumed not to affect rates of hospital readmission, inpatient mortality or long-term mortality. An overview of other assumptions can be found in the Additional file 1.

### Scenario analyses

A number of alternative scenarios were analysed. These included alternative time horizons of 60, 90 and 180 days and 1 year for all subpopulations in the model. Alternative durations of tolvaptan treatment were also analysed, as well as removing the early discontinuation of treatment rule and different hospital LOS. An SCLC population receiving tolvaptan for extended periods, typically to increase QoL in a palliative care setting, was also considered as a scenario analysis. The alternative scenarios tested are described in the Additional file 1.

### Sensitivity analysis

**Fig. 2:**
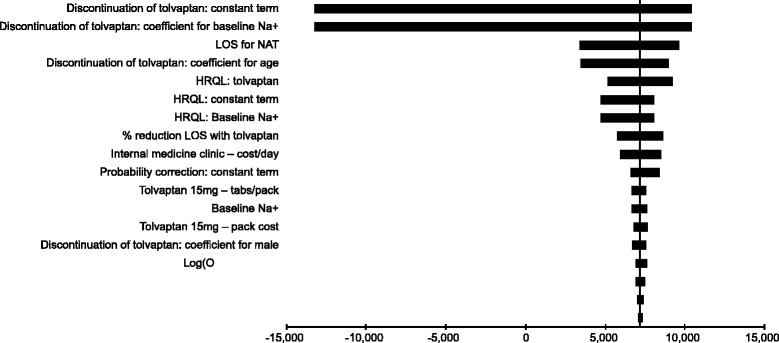
Tornado diagram showing effect of varying parameters on NMB (threshold = SEK 1.2 m). Abbreviations: HRQL, health-related quality of life; LOS, length of stay; NAT, no active treatment; NMB, net monetary benefit; OR, odds ratio; SEK, Swedish kronor

Sensitivity analysis is used to explore how changes in model parameters and assumptions alter the outcomes of the analysis. All parameters in the model were varied by the 95 % CI around the mean or, where unavailable, by ±15 %. A tornado diagram, visually representing the results (Fig. 2), was generated, in which the effect on the net monetary benefit (NMB) at a willingness-to-pay threshold SEK 1.2 million was plotted, with variables ordered by the magnitude of their effect. The NMB applies a monetary value to QALYs and is calculated by rearranging the cost-effectiveness decision rule to *λ* ⋅ Δ*E* − Δ*C* > 0 ($\lambda$, willingness to pay threshold; ΔE, change in effect; ΔC, change in cost) [25]. This approach was preferred to plotting changes to ICERs directly to ensure appropriate values were presented in the presence of negative ICERs.

## Results

Base case results for the three different populations, based on cohorts of 100,000 individually modelled patients, are presented in Table 3. Detailed results are provided in the Additional file 1. Tolvaptan was associated with reduced per patient costs (savings of SEK 2,678 to SEK 8,411[€289 to €908]) and increased incremental QALYs (0.0018 to 0.0028) versus NAT. Tolvaptan was therefore considered to be the dominant (i.e. both cost-saving and resulting in a QALY gain) strategy in all three populations.

**Table 3:** Base case results (per patient)

	NAT	Tolvaptan	Incremental
General SIADH			
Total costs	SEK 45,462	SEK 39,683	-SEK 5,779
QALYs	0.05381	0.05567	0.00186
ICER			Tolvaptan dominates
SCLC			
Total costs	SEK 372,717	SEK 364,306	-SEK 8,411
QALYs	0.21817	0.22101	0.00284
ICER			Tolvaptan dominates
Pneumonia			
Total costs	SEK 26,541	SEK 23,863	-SEK 2,678
QALYs	0.05689	0.05868	0.00179
ICER			Tolvaptan dominates

*Abbreviations: HRQL* health-related quality-of-life, *ICER* incremental cost-effectiveness ratio, *NAT* no active treatment, *QALY* quality-adjusted life-year, *SCLC* small-cell lung cancer, *SIADH* syndrome of inappropriate antidiuretic hormone secretion

The results from the scenario analyses are presented in the Additional file 1. The most important determinants of cost-effectiveness in our model were reduction in hospital LOS associated with tolvaptan (removal of this benefit resulting in ICERs of SEK >1.4 million), duration of treatment with tolvaptan (an increase in treatment duration resulting in a considerable ICER increase in the ‘all SIADH’, but not change in the SCLC and pneumonia populations) and long term treatment with tolvaptan in SCLC patients (SEK 2.9 million).

The results of univariate sensitivity analyses for the ‘general SIADH’ population are presented in Fig. 2. The most influential parameters were two of the coefficients used in the estimation of the duration of tolvaptan treatment; the constant term and baseline [Na^+^]. When these were varied between their upper and lower 95 % confidence intervals, they were able to generate NMB < SEK 0 at a willingness-to-pay threshold of SEK 1.2 million. A recent review of the literature around the value of a statistical life in Sweden suggests a willingness-to-pay threshold of SEK 1.2 million per QALY [26].

## Discussion

The present analysis assessed the cost-effectiveness of tolvaptan in three populations of patients with HN secondary to SIADH for whom the alternative treatment option is NAT. In the base case for the ‘general SIADH’ population, tolvaptan was the dominant strategy compared with NAT, as it was associated with a reduction in cost and an incremental QALY gain. Cost savings are predicted based on reductions in LOS, whilst QALY gains are a consequence of associated improvements in HRQL. Compared with NAT, tolvaptan was also the dominant strategy in the SCLC and pneumonia populations, associated with incremental cost savings and an incremental QALY gain. The results were sensitive to assumptions relating to the duration of tolvaptan treatment, LOS associated with NAT and the reduction in LOS associated with tolvaptan treatment.

The data used to calculate LOS and HRQL was obtained from the SALT I and SALT II clinical trials [4, 5]. The patients in the trials were treatment-naïve, whereas patients in the model had either failed to respond to fluid restriction or the use of fluid restriction was not suitable. Patients that fail to respond to fluid restriction may have more severe forms of SIADH and may be unable to excrete electrolyte-free water [27]. Therefore, they may respond differently to tolvaptan compared with the patients in SALT I and SALT II. Although the assumed positioning of tolvaptan in the model may be considered a limitation of the analysis, the positioning was based on Swedish guidelines for HN [1]. Furthermore, there are indications, from small case series [28, 29] that tolvaptan quickly restores serum [Na^+^] concentrations in a population consistent with that modelled (i.e. in patients with HN secondary to SIADH who have failed fluid restriction or for whom fluid restriction is unsuitable).

The model assumes a reduction in LOS of 20 % based on the overall reduction observed in patients receiving tolvaptan compared with patients receiving placebo in the SIADH population of the SALT trials [4]. This reduction was not statistically significant, and the studies were not powered to demonstrate a difference in reduction of LOS. However, a subgroup analysis of euvolaemic patients with HN in the investigator-diagnosed SIADH group, combined with patients classified as ‘other’ (all patients not meeting clinical criteria for diagnosis of heart failure or cirrhosis), demonstrated a significantly shortened LOS favouring tolvaptan (4.70 ± 3.89 versus 8.40 ± 9.67 days; *p* = 0.045; a reduction of 44 %) [4]. These preliminary findings should ideally be confirmed by clinical trials designed to evaluate this outcome. A reduction of LOS, as demonstrated in the SALT trials, is supported by preliminary data from the HN registry [30] showing that the median LOS from the start of treatment was shortest for tolvaptan (3 days) and longest for no treatment (6 days). Published data in HN relevant to the current analysis are limited, not least due to the complexity of the condition and the clinical pathway. Every effort was made to identify the most relevant data for the model, using real word data sources where possible. Sweden-specific data were used where available.

Basing model LOS data in the SCLC and pneumonia populations on evidence from the SALT trials may be considered a source of uncertainty, as the percentage of patients in the study population with SCLC or pneumonia is not known. The patients enrolled in these studies had either euvolaemic or hypervolaemic HN associated with chronic heart failure, cirrhosis or SIADH (with no condition affecting >50 % of patients) [5]. Our approach is also supported by other studies, which suggest that the mean LOS assumed for the NAT arm of the model (8 days) is reasonable for the pneumonia population [31–33]. Data for the SCLC population are scarce; Salahudeen et al. [34] report mean (SD) LOS of 10.3 (10.0) for patients with HN in a mixed US oncology population.

Patient baseline characteristics were derived from the ART study, which identifies HN patients using ICD-9/10 codes. This method of identifying patients has high specificity, but low sensitivity [35]. However, the data are specific to Sweden and include patients from all three populations modelled. The ART study is therefore the most relevant source of patient characteristics data for this analysis.

It remains unclear to what extent [Na^+^] correction or improvement is associated with improved long-term outcomes. There is some evidence that HN in hospitalised patients is associated with increased mortality (5-year data are available), with mild HN showing similar mortality rates to severe HN [36]. The model assumes no mortality benefit associated with [Na^+^] correction and does not explicitly include the benefits of treatments which may be administered to achieve [Na^+^] correction. The model assumes no residual HRQL or cost benefits associated with tolvaptan following discontinuation, and other longer-term benefits, such as any benefits of [Na^+^] correction on readmissions, are ignored by the model, and therefore cost-effectivness may be underestimated.

Another possible factor not considered in our analysis, due to the lack of data, is the possibility of overcorrection, which may occur due to unexpected diuresis after resolution of the cause of the original water retention. Overcorrection of HN can have severe neurological consequences due to demyelination occurring in the brain [37].

In modelling this condition, there was dissociation between the treatment effect (e.g. changes in [Na^+^] and HRQL. HRQL is based on reported QoL at Day 30 of SALT I and II. Transient adverse events could therefore have been missed, which would lead to an underestimation of HRQL losses. Although the assumption that 100 % of the ‘general SIADH’ population resolve their underlying condition following discharge is an over-simplification, the effect on incremental cost-effectiveness is likely to be minimal (please see Section 1.1.6 of the Additional file 1 for the underlying reasoning). Modelling HN secondary to SIADH without building several disease-specific models required a high degree of generalisation.

Published data relevant to the current analysis are limited but the best available data have been utilised from the randomised SALT trials. The main costs and benefits associated with short-term tolvaptan use are included in the model, but data limitations could lead to additional benefits not being accounted for, resulting in a possible under-estimation of cost-effectiveness. Despite a possible underestimation of cost-effectiveness, due to data limitations, the current analysis shows that tolvaptan treatment is associated with cost savings. The results of our analysis will be helpful to clinicians in the field of HN in establishing appropriate treatment strategies for patients with euvolaemic HN in the hospital setting.

## Conclusion

This is the first cost-utility analysis of tolvaptan for the treatment of HN secondary to SIADH in a European setting. Treatment with tolvaptan was associated with reduced costs and better health outcomes (measured as QALYs) compared with NAT in the ‘general SIADH’, SCLC and pneumonia populations considered in this economic evaluation. Based on the assumptions within this evaluation, tolvaptan therefore represents a cost-effective treatment option for patients with HN secondary to SIADH who have either failed to response to fluid restriction or for whom the use of fluid restriction is not suitable.

## Ethics approval and consent to participate

Not applicable. We conducted an economical assessment of data which is already in the public domain and are referenced to throughout the manuscript where relevant.

## Consent for publication

Not applicable. We conducted an economical assessment of data which is already in the public domain and are referenced to throughout the manuscript where relevant.

## Availability of data and materials

The datasets supporting the conclusions of this article are included within the article (and its additional file).

## Additional file

Additional file 1: Cost-effectiveness of tolvaptan for the treatment of hyponatraemia secondary to SIADH in Sweden. (DOCX 468 kb)

## Abbreviations

ADH: antidiuretic hormone
DES: discrete event simulation
HN: hyponatraemia
HRQoL: health related quality of life
ICER: incremental cost-effectiveness ratio
LOS: length of stay
NAT: no active treatment
QALYs: quality-adjusted life-years
QoL: quality of life
SCLC: small-cell lung cancer
SEK: Swedish kronor
SIADH: syndrome of inappropriate antidiuretic hormone secretion
TLV: the dental and pharmaceutical benefits agency
