# Supplementary material for: Cost-effectiveness of tolvaptan for the treatment of hyponatraemia secondary to syndrome of inappropriate antidiuretic hormone secretion in Sweden
Source: BMC Endocr Disord. 2016 May 16;16:22. doi: 10.1186/s12902-016-0104-z (PMC4867540; doi:10.1186/s12902-016-0104-z)
Supplement: Additional file 1: — Cost-effectiveness of tolvaptan for the treatment of hyponatraemia secondary to SIADH in Sweden. (DOCX 468 kb) [file 12902_2016_104_MOESM1_ESM.docx]

## Cost-effectiveness of tolvaptan for the treatment of hyponatraemia secondary to SIADH in Sweden

## Additional file 1

1. Data used in the model
   1. Clinical data
      1. Baseline patient characteristics: HN Registry with ART

Age and gender for each population were derived from ART as it most closely reflects the patient population considered in Sweden. Baseline sodium levels were not available from ART and are, therefore, taken from the HN Registry. This was preferred to SALT I & II as it is believed to better reflect ‘real-world’ treatment of HN. Hypothetical patients were generated via random sampling of the ART study patient characteristics; age was assumed to be normally distributed, gender was assumed to follow a binomial distribution and baseline sodium level was normally distributed. Patient characteristics are presented in Table 1.

Table 1: Baseline characteristics for model base case

| Characteristic | Age (SD)^†^ | Male | [Na^+^] (SD)^‡^ | Sources |
| --- | --- | --- | --- | --- |
| ‘All SIADH’ | 69.8 (16.76) | 33% | 124.6 (5.8) | ART^§^ Table 1  HN Registry |
| SCLC | 65.5 (16.76) | 31% | 123.1 (6.0) | ART^§^ Table 19  HN Registry [1] |
| Pneumonia | 69.3 (16.76) | 43% | 124.6 (5.8)^¶^ | ART^§^ Table 50  HN Registry |

Abbreviations: ART, The Assessment of epidemiology, patient characteristics and outcomes Related To patients with hyponatraemia/SIADH in Sweden; HN, hyponatraemia; Na+, sodium; SCLC, small-cell lung cancer; SD, standard deviation; SIADH, syndrome of inappropriate antidiuretic hormone secretion.

†Drawn from a normal distribution with a minimum age of 18 and maximum age of 100 applied. All populations assumed to have standard deviation=16.76

‡ Drawn from a normal distribution with a maximum baseline sodium of 129.9 applied
§ Analysis of ART study
¶ Assumed to be the same as the ‘all SIADH’ population

- - 1. Probability of sodium correction

Sodium correction is defined as [Na^+^] >135 mmol/L at day 4, in line with a pre-defined endpoint in SALT I & II [2]. The threshold of [Na^+^] >135 mmol/L is similar to that considered by many studies that reported a definition of [Na^+^] correction [3-6]^[[1]](#endnote-1)^.

- - - - 1. Base case analysis: HN Registry with SALT I & II

The HN Registry [unpublished observations] represents ‘real-world’ efficacy of tolvaptan in a position in the treatment pathway that was believed to be more closely aligned to where tolvaptan is used in Sweden than SALT I & II. SALT I & II, however, remains the only source of randomised, controlled evidence available to make a comparison of tolvaptan and NAT. A hybrid approach is therefore adopted, in which relative efficacy from SALT I & II is applied to a baseline probability of sodium correction from the HN Registry.

Although evidence from the HN Registry permits the probability of correction for individuals receiving no treatment, the observational nature of the study means that such a comparison (tolvaptan versus NAT) may be adversely affected by selection bias (because less severe patients may be less likely to be treated). The relative efficacy of tolvaptan versus NAT is, therefore, derived from SALT I & II, the only Phase III randomised controlled trials (RCTs) to consider this comparison. The SIADH analysis presented by Verbalis et al [7] is used to maintain consistency with the populations evaluated by this analysis.

The odds ratio of achieving [Na^+^] correction at day 4 for the tolvaptan arm versus the NAT arm is estimated as 11.5 (95% CI: 4.2–32.1) from data presented by Verbalis et al [7].^[[2]](#endnote-2)^ This is applied to the odds of [Na^+^] correction for NAT (estimated using the regression model in Table 2) in order to provide the probability of [Na^+^] correction for tolvaptan.

In order to allow for patient heterogeneity, the baseline probability of response is estimated using a binary logistic regression based on data from the HN Registry. Baseline characteristics (age, gender, baseline [Na^+^]) were controlled for. The availability of patient numbers by subgroup (SCLC and pneumonia) was insufficient to support the estimation of population-specific regression models; the regression model for the overall population is, therefore, assumed for all populations modelled (‘all SIADH’, SCLC, and pneumonia). This is a limitation of the analysis; however, since patient characteristics are permitted to vary by population, this does not result in an equal probability of correction between populations.

Table 2 presents the results of binary logistic regression for [Na^+^] correction. Tolvaptan is associated with a statistically significant (log) odds ratio of achieving correction (p<0.0001); however, this data point is not used in the analysis in preference to randomised evidence from SALT I & II, as described above.

Table 2: Binary logistic regression of achieving correction in [Na^+^] at day 4 [unpublished observations, analysis of HN Registry]

|  | Coef. | Std. Err. | Wald | p-value | OR | 95% CI | |
| --- | --- | --- | --- | --- | --- | --- | --- |
| Age (≤50 ref) |  | | | | | | |
| 51-64 | -0.443 | 0.455 | 0.951 | 0.329 | 0.642 | 0.263 | 1.565 |
| 65-74 | -0.364 | 0.474 | 0.588 | 0.443 | 0.695 | 0.275 | 1.76 |
| ≥ 75 | -1.017 | 0.45 | 5.097 | 0.024 | 0.362 | 0.15 | 0.874 |
| Male | 0.601 | 0.318 | 3.561 | 0.059 | 1.824 | 0.977 | 3.405 |
| Baseline [Na^+^] | 0.047 | 0.037 | 1.626 | 0.202 | 1.048 | 0.975 | 1.126 |
| Tolvaptan vs. NAT (logOR) | 2.021^†^ | 0.339 | 35.47 | <.0001 | 7.549 | 3.881 | 14.682 |
| _cons | -7.025 | 4.674 |  |  |  |  |  |

Abbreviations: CI, confidence interval; coef, coefficients; logOR, log odds ratio; OR, odds ratio; Std. Err., standard error.
† The base case analysis does not use this value (as discussed in the text above); instead, this value is replaced with efficacy data from SALT I & II in the estimation of the probability of response for tolvaptan. This provides a log odds ratio of [Na^+^] correction of approximately log (11.5) = 2.44 for comparison.

- - 1. Duration of tolvaptan treatment
       - 1. Base case analysis for ‘all SIADH’: HN Registry

The duration of tolvaptan treatment in the ‘all SIADH’ population is estimated from the HN Registry. An episode of care is defined as “concurrent daily administrations of tolvaptan with no more than 72hrs between any 2 doses”. In order to estimate the duration of tolvaptan treatment, the duration of each treatment episode of care is summed together, by patient, during a hospital admission to provide a total duration of tolvaptan treatment.

Table 3: Baseline characteristics of tolvaptan monotherapy patients from HN Registry

| Variable | Obs. | Mean | SD |
| --- | --- | --- | --- |
| Age (years) | 60 | 61.55 | 15.4 |
| Male (%) | 61 | 42.6% | - |
| Baseline [Na^+^] | 61 | 124.6 | 5.8 |
| European (%) | 61 | 78.69% | - |

Abbreviations: HN, hyponatraemia; Obs., observations; SD., standard deviation

Figure 1 presents observed treatment duration from 210 patients in the HN Registry.

Figure 1: Kaplan-Meier plot of tolvaptan treatment duration from HN Registry

Model selection is based on the methods described by the National Institute for Health and Care Excellence (NICE) Decision Support Unit (DSU) [8]. Specifically, five alternative parametric model specifications were estimated and model selection was based on minimisation of the Akaike Information Criterion (AIC). The generalised gamma distribution could not be estimated using maximum likelihood with the available data. Table 4 presents model goodness-of-fit statistics for alternative survival distributions. The lognormal distribution offers the best model fit based on both the AIC and the Bayesian Information Criterion (BIC) [9]. Note the number of observations is 54 because of exclusions due to missing data points.

Table 4: Model goodness-of-fit statistics for duration of tolvaptan treatment from HN Registry

| Model | Obs | ll(null) | ll(model) | df | AIC | BIC |
| --- | --- | --- | --- | --- | --- | --- |
| exponential | 54 | -87.86342 | -69.27753 | 5 | 148.5551 | 158.5 |
| weibull | 54 | -85.71248 | -64.48176 | 6 | 140.9635 | 152.8974 |
| gompertz | 54 | -78.83504 | -68.77021 | 6 | 149.5404 | 161.4743 |
| lognormal | 54 | -71.02982 | -62.03398 | 6 | 136.068 | 148.0019 |
| loglogistic | 54 | -69.66127 | -63.67736 | 6 | 139.3547 | 151.2886 |

Abbreviations: AIC, Akaike information criterion; BIC, Bayesian information criterion; df, degrees of freedom; ll, log-likelihood; obs, observations.

Table 5 presents the selected lognormal regression model.

Table 5: Lognormal regression model for duration of tolvaptan treatment from HN Registry

|  | Coef. | Std. Err. | z | P>z | 95% CI | |
| --- | --- | --- | --- | --- | --- | --- |
| Age | .0011677 | .0069154 | 0.17 | 0.866 | -.0123862 | .0147216 |
| Male | .0469536 | .2166024 | 0.22 | 0.828 | -.3775793 | .4714866 |
| Baseline [Na^+^] | -.0502804 | .0224336 | -2.24 | 0.025 | -.0942494 | -.0063114 |
| Correction^†^ | .0226724 | .0057299 | 3.96 | 0.000 | .0114419 | .0339028 |
| _cons | 6.912861 | 2.935357 | 2.36 | 0.019 | 1.159666 | 12.66606 |
| /ln_sig | -.2701612 | .096225 | -2.81 | 0.005 | -.4587588 | -.0815636 |
| sigma | .7632564 | .0734444 |  |  | .6320677 | .9216741 |

Abbreviations: CI, confidence interval; coef., coefficient; Std. Err., standard error.

† Defined as [Na^+^] ≥135 mmol/L at day 4

- - - - 1. Base case analysis for SCLC and pneumonia populations: Expert clinical opinion

Sample size considerations meant that reliable duration of treatment data for SCLC and pneumonia populations were not available from the HN Registry. However, clinical expert opinion suggested that the duration of tolvaptan treatment is highly population specific. Therefore, the HN Registry estimates used in the ‘all SIADH’ population were not considered appropriate for subgroups.

Mean duration of tolvaptan treatment for a single admission in the SCLC population was assumed to be 4 days based on the median duration of tolvaptan treatment reported by Petereit et al [10]. This is supported by analysis of 9 patients with pulmonary tumours in the HN Registry, who had a mean duration of exclusive tolvaptan monotherapy treatment of 5.3 days [unpublished observations, analysis of HN Registry].

Mean treatment duration for a single HN episode in the pneumonia population was assumed to be 3 days, based on the mid-point of values provided by clinical expert opinion. Of patients selected for inclusion within the analysis of HN Registry data, no individuals had a diagnosis of pneumonia at admission. However, analysis of 8 patients who did not exclusively receive tolvaptan monotherapy (i.e. patients who may have experienced multiple HN treatments during an admission) with a diagnosis of pneumonia had a mean duration of tolvaptan treatment of 2.6 days [unpublished observations, analysis of HN Registry].

- - - - 1. Scenario analysis: PASS

Another potential source of treatment duration data is the tolvaptan Post-Authorisation Safety Study (PASS), which is included within scenario analyses. PASS is a prospective, multicentre, multinational, observational study to document the utilisation of tolvaptan and to collect information on its safety when used in routine medical practice. Preliminary data from PASS shows a high degree of variation in treatment duration, with a large number of patients completing treatment episodes quickly and a minority continuing to use tolvaptan for longer durations of treatment (Figure 2). Parametric survival distributions were fitted to these data, and a generalised gamma distribution selected based on the minimisation of the AIC can be selected within the model [unpublished observations, Otsuka].

Figure 2: Tolvaptan treatment duration from PASS


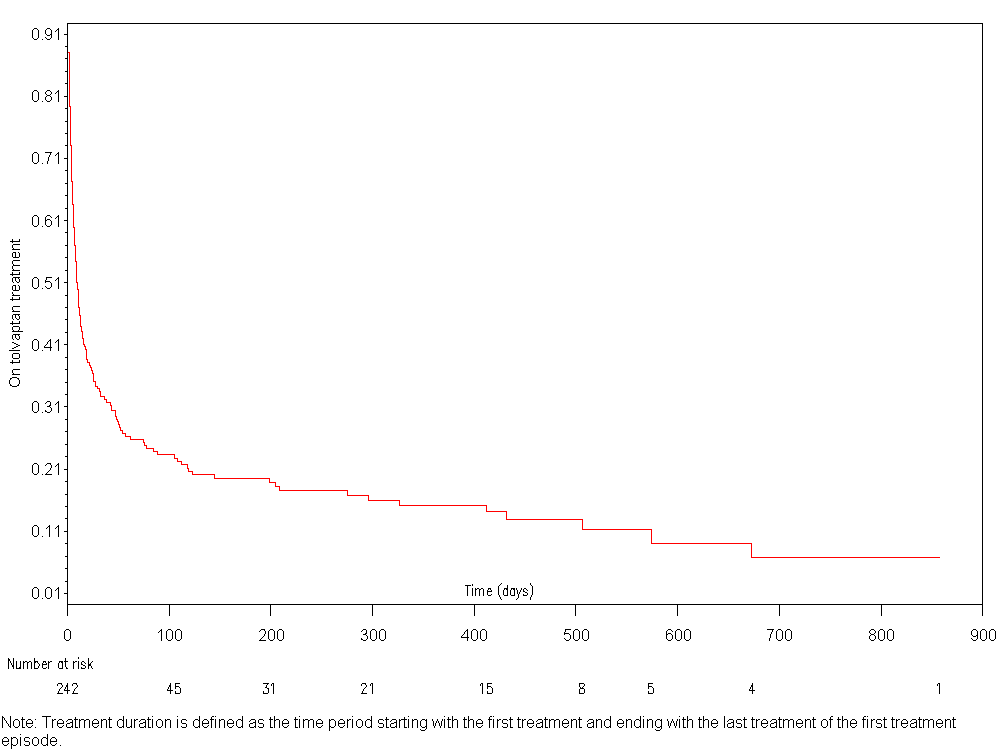


- - 1. Early tolvaptan treatment discontinuation rule

In addition to duration of tolvaptan treatment as described in Duration of tolvaptan treatment, it is further assumed that patients who fail to achieve correction will be subject to an early tolvaptan treatment discontinuation rule in which patients discontinue tolvaptan treatment at day 2. Clinical expert opinion confirmed that individuals who failed to respond to tolvaptan would discontinue treatment with tolvaptan. Clinical expert opinion suggested that a range of 2-4 days was plausible within clinical practice. The model considers [Na^+^] correction at day 4 (Probability of sodium correction), and it is therefore assumed that clinicians would discontinue tolvaptan treatment *following the observation of low rates of change in [Na^+^]* *at day 2 which would ultimately be associated with a failure to achieve correction of [Na^+^].* In other words, although the model uses [Na^+^] at day 4 to determine whether the early tolvaptan discontinuation rule is applied, the early tolvaptan discontinuation rule is assumed to be applied at day 2, following clinical observation that [Na^+^] has not changed sufficiently such that correction at day 4 is possible.

Scenario analysis also considers a removal of the early tolvaptan treatment discontinuation rule.

- - 1. Length of stay

Hospital length of stay is a key determinant of resource use during inpatient admissions and is, therefore, the main resource use cost in the analysis. Specific costs for items such as chemotherapy in the SCLC population and sodium monitoring are considered separately.

- - - - 1. The effects of HN on hospital LOS

Hyponatraemia has been shown to be associated with increased hospital LOS in multiple studies [4, 11-21], although this observation has not been universal [22] and an inverse relationship between baseline serum [Na^+^] and LOS has also been observed in some studies [3, 23-25].

Nair et al [15] analysed 342 hospitalised patients with community-acquired pneumonia in the US. The authors observed a mean (SD) LOS of 8.66 (4.3) days in patients with HN and 6.4 (3.9) days in patients without HN (p<0.001). Similarly Zilberberg et al [21] reported a retrospective analysis of patient discharges with a principal discharge diagnosis of pneumonia in the US and observed a LOS of 7.6 (5.3) days and 7.0 (5.2) days for patients with and without HN, respectively (p<0.001). Wald et al [20] retrospectively analysed hospital discharges (mixed aetiologies) from a single US centre. Hospital-acquired HN was associated with a 64% (95% CI: 60%-68%) adjusted increase in LOS. The authors concluded that all forms of HN are independently associated with in-hospital mortality and increased resource consumption.

The effect of [Na^+^] correction on hospital LOS is not fully understood. No data within the licensed indication for tolvaptan was identified. Hackworth et al [13] observed a numerically lower (but not statistically significant) LOS for resolved versus persistent HN in a retrospective review of outcomes following orthotopic liver transplantation (21 versus 27 days, respectively).

- - - - 1. Base case analysis: SALT I & II

In order to inform the LOS and the reduction associated with tolvaptan, the percentage reduction in hospital LOS observed from SALT I & II (SIADH) populations was selected. Post-hoc analysis demonstrated a reduction in mean LOS of 20% with tolvaptan (calculated from data presented in Table 6) in the SIADH population with [Na^+^] <130 mmol/L. This analysis did not show a statistically significant difference in LOS, but was not sufficiently powered to do so^[[3]](#endnote-3)^. This population was preferred to the SALT I & II whole SIADH population presented by Verbalis et al [7] because clinical expert opinion suggested that in Sweden individuals treated with HN secondary to SIADH would be expected to have a baseline [Na^+^] < 130 mmol/L. The baseline characteristics of the modelled population from the HN Registry also have a [Na^+^] of approximately 125 mmol/L (Baseline patient characteristics: HN Registry with ART).

Table 6: Hospital LOS by study arm, SALT-1 and SALT-2, <130 mmol/L serum [Na^+^] SIADH population

|  | N | Mean hospital LOS | Median hospital LOS | SD | Minimum hospital LOS | Maximum hospital LOS | 95% CI around difference | | p-value |
| --- | --- | --- | --- | --- | --- | --- | --- | --- | --- |
| Tolvaptan | 25 | 6.40 | 4 | 8.39 | 2.00 | 43.00 | -6.87 | 3.87 | 0.5759 |
| Placebo | 20 | 8.00 | 4 | 9.31 | 2.00 | 33.00 |  |  |  |

Abbreviations: CI, confidence interval; LOS, length of stay; SD, standard deviation.
Source: [post-hoc analysis of SALT-1 and SALT-2].

Further analysis of hospital LOS data in the SIADH population was performed by Verbalis et al [7] and is summarised here. Analysis by severity of HN indicated that there were differences in LOS depending on baseline [Na^+^]. Patients with mild HN (defined as a [Na^+^] ≥130 mmol/L) had shorter LOS (tolvaptan=3.00 ± 1.24 vs. placebo= 4.61 ± 6.20 days, p=0.212) than patients with [Na^+^] <130 mmol/L (tolvaptan=6.40 ± 8.39 vs. placebo=8.00 ± 9.31 days, p=0.576). Although neither of these analyses reached statistical significance, analysis of the investigator-diagnosed SIADH group combined with patients classified as ‘other’ (i.e. all patients not meeting clinical criteria for diagnosis of heart failure or cirrhosis) demonstrated a significantly shortened LOS favouring tolvaptan in the [Na^+^] <130 mmol/L HN group (tolvaptan=4.70 ± 3.89; placebo= 8.40 ± 9.67 days; p=0.045).

- - 1. Resolution of underlying cause of HN secondary to SIADH

Expert clinical opinion highlighted the importance of the resolution of underlying morbidities in determining the HRQL of an individual following discharge from the inpatient setting. The probability of such resolution is believed to differ by population. In the pneumonia population, expert opinion suggested most patients would receive successful treatment for their pneumonia infection and the underlying cause of HN (SIADH) is, therefore, resolved. This was also conservatively applied as a base case assumption in the ‘all SIADH’ cohort. A 100% rate of resolution is, therefore, assumed for both the ‘all SIADH’ and pneumonia populations, without having clinical data to provide a more accurate % resolution in each population and the clinical characteristics that would be associated with a specific non-resolution outcome. This assumption has minimal impact on the overall conclusions of the analysis, as it is applied to both arms of the model.

Within SCLC cohorts, however, List et al [26] observed that HN secondary to SIADH resolved with cytotoxic therapy in 31 of 35 patients (88%). Following tumour progression, HN recurred in 70% of patients [26]. Tai et al [27] observed 12/14 patients (86%) with HN secondary to SIADH at initial presentation achieved a normal [Na^+^] after treatment with combination chemotherapy. In the SCLC population considered within this analysis (which was assumed to be predominantly early stage patients undergoing chemotherapy), it is assumed that 88% of patients would experience resolution of HN secondary to SIADH at hospital discharge^[[4]](#endnote-4)^. In practice, this assumption has minimal impact on the overall conclusions of the analysis, as it is applied to both arms of the model. It is of note that these assumptions represent a simplification of these real-world features; chemotherapy-related HN resolution (or correction) is used as a proxy for the resolution of SIADH.

- - 1. Hospital readmission
       - 1. The effects of hyponatraemia on hospital readmission

Three studies that considered hospital readmission rates were identified [11, 28, 29].

Scherz et al [29] examined readmission in HN patients with acute pulmonary embolism. The authors retrospectively considered 13,728 patient discharges with a primary diagnosis of pulmonary embolism in the US. The probability of readmission was increased for patients with [Na^+^] 130‑135 mmol/L at initial presentation (adjusted OR, 1.28; 95% CI: 1.12–1.46) and [Na^+^] < 130 mmol/L (adjusted OR, 1.44; 95% CI: 1.02–2.02).

Borenstein et al [11] report that HN was associated with an increased 30-day readmission risk (OR=3.49; 95% CI: 1.3–9.35) in a prospective cohort study of 214 hospitalised US Medicare beneficiaries.

Gheorghiade et al [28] examined 6-month post-discharge death or rehospitalisation, following an RCT of pulmonary artery catheter plus clinical assessment versus clinical assessment alone, in heart failure (HF) patients. After controlling for baseline variables and clinical response, the authors found that patients with persistent HN had an increased risk of death or HF rehospitalisation (62% versus 43%; HR, 1.52) (p=0.03).

Therefore, although the literature suggests HN at patient presentation is associated with increased risk of readmission, it is not yet established whether correction of [Na^+^] is associated with reduced readmission rates for the patient populations under consideration within this evaluation.

- - - - 1. Base case analysis: ART

Time to readmission is taken from ART (from the relevant ‘all SIADH’, SCLC and pneumonia patient populations), as the only Swedish-specific readmission data identified. The model uses a two-step process, firstly determining whether a patient was ever readmitted before secondly generating a time to readmission based on a Weibull distribution if readmission occurs. Table 7 presents the parameters used within the model; predicted versus observed fits for the selected Weibull distributions are presented in Figure 3 to Figure 5.

In the absence of alternative appropriate data sources, no benefit is applied to the risk of readmission for corrected versus persistent HN. As a sensitivity analysis, a hazard ratio for corrected versus persistent HN is approximated by the relative risk from Gheorghiade et al [28], and applied to the baseline risk of readmission from ART. The approximated hazard ratio is estimated as 25%/62%=0.403.

Table 7: Readmission parameters in model from ART [unpublished observations]

| Population | N total | % censored | Scale | Scale SE | Shape | Shape SE |
| --- | --- | --- | --- | --- | --- | --- |
| All SIADH | 1619 | 22.174 | 137.34 | 0.2180 | 0.5844 | 0.00108 |
| SCLC | 67 | 11.94 | 62.5 | 0.8277 | 0.5713 | 0.00953 |
| Pneumonia | 135 | 24.444 | 110.6 | 0.7679 | 0.6772 | 0.00514 |

Abbreviations: SCLC, small-cell lung cancer; SE, standard error; SIADH, syndrome of inappropriate antidiuretic hormone secretion.

Figure 3: ‘All SIADH’ population; fitted Weibull (brown) vs. observed in ART (blue) [unpublished observations]


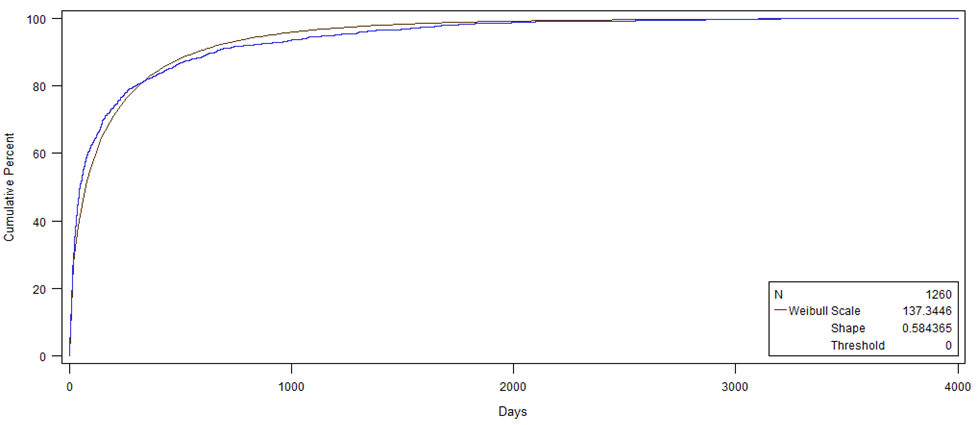


Abbreviations: ART, the Assessment of epidemiology, patient characteristics and outcomes Related To patients with hyponatraemia/SIADH in Sweden; SIADH, syndrome of inappropriate antidiuretic hormone secretion.

Figure 4: SCLC population; fitted Weibull (brown) vs. observed in ART (blue) [unpublished obervations]


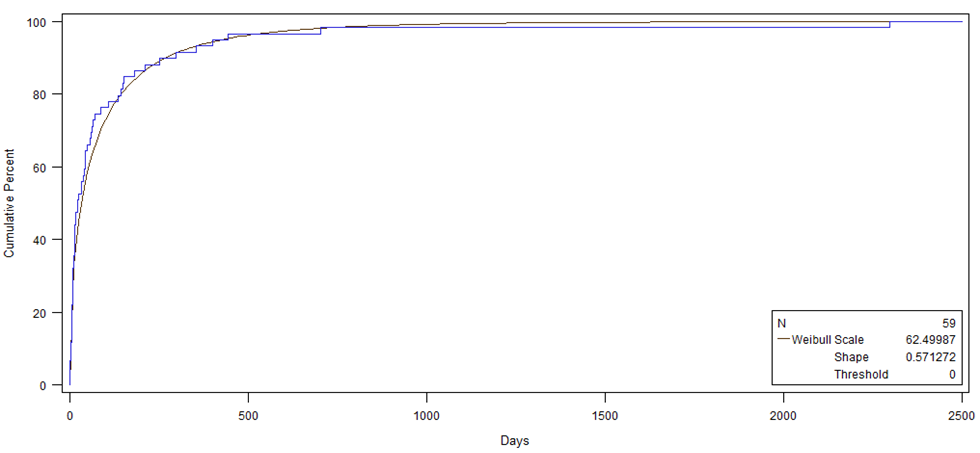


Abbreviations: ART, the Assessment of epidemiology, patient characteristics and outcomes Related To patients with hyponatraemia/SIADH in Sweden; SCLC, small cell lung cancer.

Figure 5: Pneumonia population; fitted Weibull (brown) vs. observed in ART (blue) [unpublished observations]


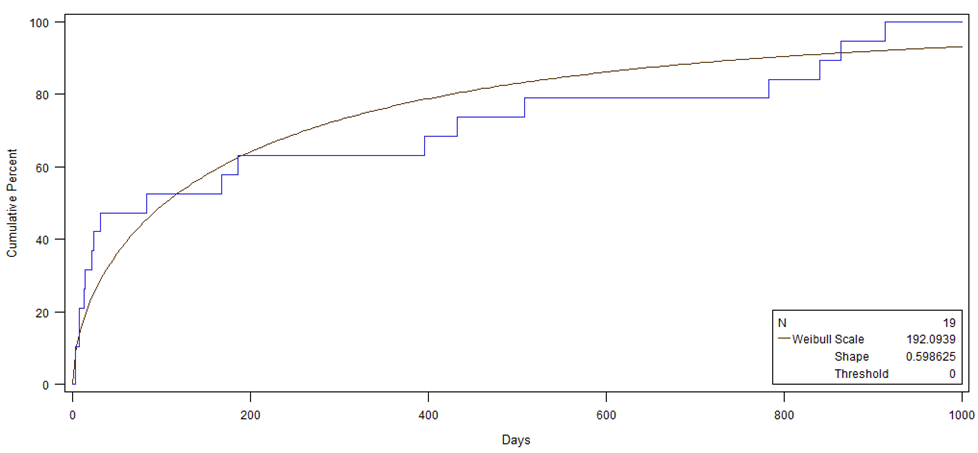


Abbreviations: ART, the Assessment of epidemiology, patient characteristics and outcomes Related To patients with hyponatraemia/SIADH in Sweden.

- - 1. Inpatient mortality
       - 1. The effect of hyponatraemia on inpatient mortality

Hyponatraemia has been demonstrated to be a negative prognostic factor for inpatient mortality in several studies [15, 18-20, 22, 30-32]. The direction of causal mechanisms between HN and survival are not well understood but Nair et al [15], in an observational study of community-acquired pneumonia, concluded it is likely that HN was a marker for greater pneumonia disease severity and that the association with mortality reflects this relationship.

The effect of [Na^+^] correction on inpatient mortality is unclear but Hoorn et al [33] considered 38 patients with severe HN ([Na^+^] <125 mmol/L) on admission and 36 patients with hospital-acquired severe HN in a hospital-wide cohort study^[[5]](#endnote-5)^. Nineteen patients (26%) from both groups of patients were not treated for HN and this was associated with a higher mortality rate (7 out of 19 versus 7 out of 55, p=0.04).

- - - - 1. Base case analysis: ART

In the presence of this uncertainty, it is conservatively assumed that [Na^+^] correction has no impact on inpatient mortality. Inpatient mortality for all arms of the model is taken from ART and is reported in Table 8.

Table 8: Inpatient mortality assumptions

|  | All SIADH | SCLC | Pneumonia |
| --- | --- | --- | --- |
| Probability inpatient mortality | 2.2% | 6.2% | 4.4% |

Abbreviations: SCLC, small-cell lung cancer; SIADH, syndrome of inappropriate antidiuretic hormone secretion.

- - 1. Long-term survival
       - 1. The effects of HN on long-term survival

Hyponatraemia has been demonstrated to be a prognostic factor indicating a poor survival prognosis in several longer-term studies [3, 5, 6, 28, 34-37].

Gill et al [35] compare 104 patients with HN ([Na^+^] <135 mmol/L) and 104 randomly chosen normonatraemic patients. The study included mixed aetiologies but SIADH was reported in 10% of patients. The authors report overall mortality of 27% in the HN group versus 9% in the normonatraemic group (p=0.009) and observe increased mortality in those with [Na^+^] <120 mmol/L versus those with [Na^+^] >120 mmol/L (odds ratio 1.57, p<0.001).

Doshi et al [38] conduct a retrospective analysis of prospectively collected data in 3,357 US patients with cancer. HN ([Na^+^] < 135 mEq/L) was noted in 47% of admissions. Hazard ratios for 90-day mortality in mild, moderate, and severe HN were 2.04 (p< 0.01); 4.74 (p<0.01), and 3.46 (p=0.04), respectively. The authors conclude that HN was associated with longer hospital stay and higher mortality in patients with cancer.

Hermes et al [6] consider the clinical significance of HN ([Na^+^] < 135 mmol/L) in an unselected contemporary patient population with SCLC in Germany. HN was present in 18.9% of cases. HN was observed to be associated with significantly shorter survival (SCLC all patients: 9.0 versus 13.0 months, p<0.001, limited disease SCLC: 9.0 versus 17.0, p=0.050, extensive disease SCLC: 9.0 versus 10.0, p=0.135). Following multivariate adjustment for confounding factors, HN was an independent predictor of mortality in patients with extensive disease and limited disease SCLC.

The role of [Na^+^] correction in improving survival is less clear. Hansen et al [5] considered a retrospective cohort of SCLC patients. Those who did not fully regain normal values of [Na^+^] had worse survival compared with the patients who did (p=0.027) and, in a Cox multivariate analysis, not obtaining a normal value of [Na^+^] was a statistically significant prognostic factor leading to poor survival.

- - - - 1. Base case analysis: ART

Long-term survival is based on Swedish life-tables [39] for the ‘all SIADH’ and pneumonia populations. Survival for individuals with SCLC is notably poorer than the general population; survival distributions are, therefore, fitted to the observed Kaplan-Meier plots from ART. The Kaplan-Meier plots were digitised to create a pseudo-patient-level dataset [40]. Model selection was based on comparison of the AIC across the six standard survival distributions and a Gompertz distribution was ultimately selected. Figure 6 presents the observed versus predicted estimates of survival.

Figure 6: Observed vs. predicted survival in SCLC cohort


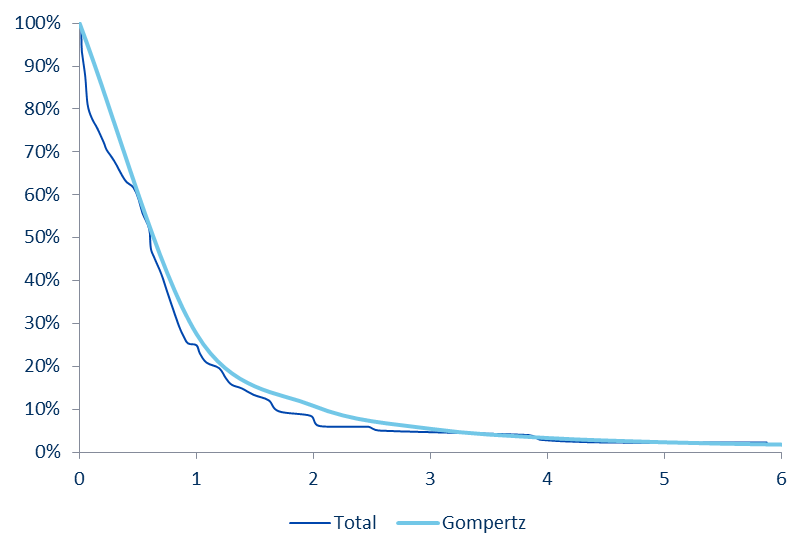


Table 9 presents the Gompertz regression parameters.

Table 9: Gompertz regression for survival in SCLC

|  | Coef. | Std. Err. | z | P>z | [95% CI] | |
| --- | --- | --- | --- | --- | --- | --- |
| _cons | 0.40856 | 0.007 | 57.250 | 0.000 | 0.395 | 0.423 |
| /gamma | -0.32137 | 0.006 | -52.370 | 0.000 | -0.333 | -0.309 |

Abbreviations: CI, confidence interval; coef., coefficient; Std. Err., standard error.

- 1. Utility data
     1. Overview of assumptions

The application of utility values during treatment is assumed to be dependent on which treatment a patient receives (NAT or tolvaptan), as this was observed to be a much better predictor of HRQL at day 30 in SALT I & II than [Na^+^] correction. Individuals are assumed to have a change in HRQL associated with treatment (tolvaptan or NAT) which changes linearly from baseline to day 4, from which point HRQL is assumed constant until the end of treatment or discontinuation of tolvaptan (whichever is of longest duration). In the base case analysis, individuals are assumed to experience the benefits of treatment for the duration of treatment or until discharge (whichever is later). As the only source of HRQL data available for which utility values required to construct QALYs are available, Short Form 12 questionnaire (SF-12) data from SALT I & II were converted to EuroQol 5-dimension (EQ-5D UK tariff) scores using a publicly-available algorithm by Gray et al [41]. It is also noted that the analysis implicitly contains the HRQL consequences of adverse events associated with tolvaptan, though it is acknowledged that by using estimates from day 30 of SALT I & II, transient adverse events may be missed within the analysis.

Following the end of tolvaptan treatment (or discharge), clinical expert opinion suggested that HRQL would be determined by whether the underlying cause of HN had been resolved or not. In the absence of HRQL data for patients with resolved underlying conditions, it is assumed that HRQL in patients with the resolution of underlying conditions is the same as that experienced by tolvaptan patients (Figure 7).

In patients who are not considered resolved following the end of tolvaptan treatment (or discharge), it is assumed that HRQL would return to baseline serum [Na^+^] of treatment discontinuation (tolvaptan) or discharge (Figure 8). In both scenarios the incremental QALY gain associated with tolvaptan is given by the area ‘A’.

Figure 7: HRQL scenario, individuals considered resolved


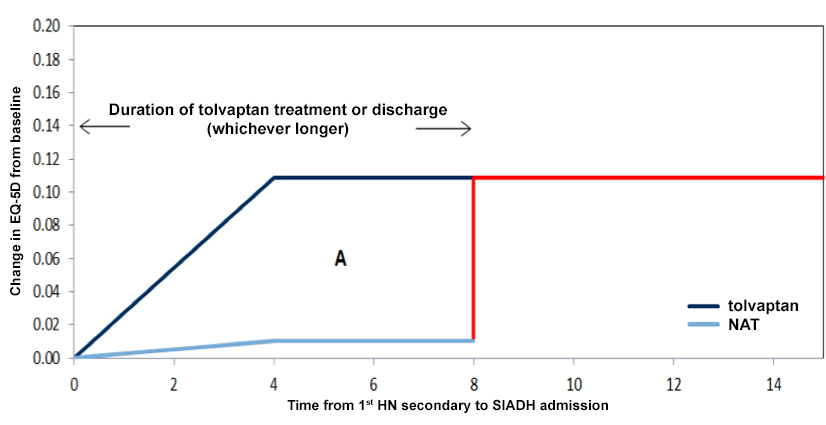


Figure 8: HRQL scenario, individuals not considered resolved


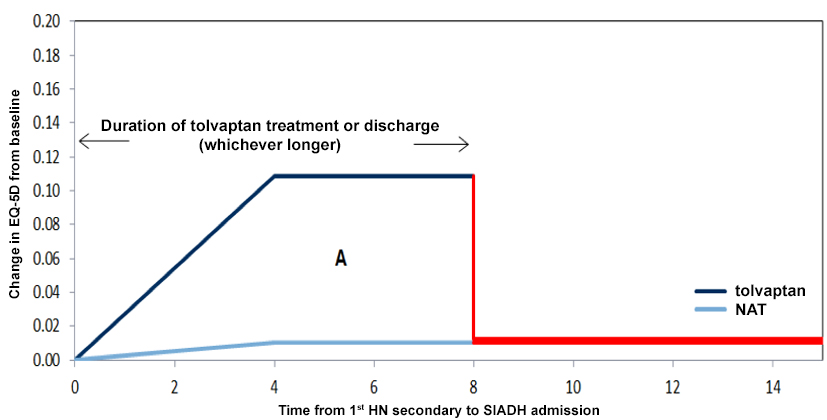


The proportion of patients considered resolved (or not) are detailed in Resolution of underlying cause of HN secondary to SIADH; however, the model is also able to consider a time-to-resolution event for patients who are not considered resolved at discharge but who would subsequently go on to resolution. In the absence of data to inform such an assumption, this model feature is not used in the base case analysis.

- - 1. Estimating change in EQ-5D

Clinical studies of tolvaptan to date have not included generic preference-based measures of HRQL. In order to allow estimates of HRQL, responses to the SF-12 from SALT I & II were mapped to EQ-5D scores (hereafter referred to as simulated EQ-5D) using a publicly-available mapping algorithm published by Gray et al [41]. This algorithm utilises the UK EQ-5D tariff by Dolan [42]. Patients from SALT I & II with heart failure and who had undergone liver transplantation were excluded from the HRQL analysis, in order to reflect the population considered within the model most closely. Unfortunately, many observations were missing at intermediary time points (Table 10); the analysis, therefore, uses data observed at day 30. This time point is used as a proxy to represent the maximum change in EQ-5D achievable for tolvaptan and NAT model arms. It is assumed that this level is achieved after 4 days (i.e. not before the population is considered as having achieved [Na^+^] correction; Probability of sodium correction) and maintained until discontinuation or discharge (whichever is greater; see Figure 7 and Figure 8). Table 10 presents simulated EQ-5D availability at the time points specified within the protocol.

Table 10: Available SF-12 data at various time points from SALT I & II population

| Time of Visit | Available SF-12 Data (no. of patients) |
| --- | --- |
| Day 1 | 180 |
| Day 2 | 3 |
| Day 3 | 0 |
| Day 4 | 1 |
| Day 7 | 78 |
| Day 14 | 86 |
| Day 21 | 4 |
| Day 30 | 167 |
| 7 day follow-up | 75 |

Source: [43].

In the primary analysis ordinary least squares regression is used to estimate the change in EQ-5D from baseline at day 30, initially as a function of baseline characteristics (age, gender, baseline [Na^+^] and baseline EQ-5D). Further regression models include indicators for treatment arm (tolvaptan or placebo) and/or correction, defined as [Na^+^] > 135 mmol/L at day 4. Model selection is based on adjusted R-squared. Table 11 presents the results of considered models and Figure 9 and Figure 10 present diagnostics for the preferred model (Model (2) within Table 11).

The indicator variable for correction at day 4 is not statistically significant and its inclusion results in a small decrease in the adjusted R-squared value. Both regression models including treatment arm as a covariate show a statistically significant increase in change in EQ-5D for tolvaptan. The tolvaptan EQ-5D utility benefit used in the analysis was therefore 0.0980 (Table 11). The use of treatment-specific HRQL also means that treatment-specific events associated with changes in HRQL (such as adverse events) will be implicitly included within the analysis.

Figure 9 and Figure 10 present diagnostic plots. Residuals appear well distributed and there were no obvious trends.

Table 11: Regression Table for Change in EQ-5D at Day 30. Coefficients (standard errors)

|  | **Model 1**  **Change in EQ-5D**  **At Day 30** | **Model 2**  **Change in EQ-5D**  **At Day 30** | **Model 3**  **Change in EQ-5D**  **At Day 30** | **Model 4**  **Change in EQ-5D**  **At Day 30** |
| --- | --- | --- | --- | --- |
| Age | -0.00141 (0.00158) | -0.00111 (0.00157) | -0.00132 (0.00164) | -0.00104 (0.00162) |
| Male (Indicator) | 0.0385 (0.0455) | 0.0173 (0.0460) | 0.0371 (0.0466) | 0.0160 (0.0470) |
| Baseline [Na^+^] | -0.000807 (0.00624) | -0.000936 (0.00616) | -0.000537 (0.00671) | 0.000832 (0.00665) |
| Baseline EQ-5D | -0.713^***^ (0.0759) | -0.711^***^ (0.0750) | -0.715^***^ (0.0770) | -0.710^***^ (0.0760) |
| Tolvaptan Arm (Indicator) |  | 0.0980^*^ (0.0443) |  | 0.107^*^ (0.0473) |
| Corrected at Day 4 (Indicator) |  |  | -0.00267 (0.0480) | -0.0350 (0.0495) |
| Constant | 0.662 (0.830) | 0.622 (0.820) | 0.624 (0.887) | 0.398 (0.881) |
| Observations | 164 | 164 | 161 | 161 |
| Adjusted *R*^2^ | 0.348 | 0.364 | 0.343 | 0.361 |

t statistics in parentheses

* p<0.05, ** p<0.01, *** p<0.001

Figure 9: Residuals against Fitted Values for Model 2 (Change in EQ-5D at Day 30)

Figure 10: Predicted Against Actual Values for Model 2 (Change in EQ-5D at Day 30)

- - 1. Baseline EQ-5D

The mapping analysis provides baseline simulated EQ-5D for a general HN secondary to SIADH population of 0.58 (S.D: 0.29) and this is used to represent the baseline EQ-5D score for the ‘all SIADH’ population. The average of this population is not considered representative of the SCLC and pneumonia populations; literature sources were therefore sought. Baseline utility scores are estimated in these populations by subtracting the tolvaptan effect observed in Table 11 from literature sources [44, 45] to provide an approximate estimate for each population ‘with HN’. This results in estimates of 0.61^[[6]](#endnote-6)^ and 0.73^[[7]](#endnote-7)^ in SCLC and pneumonia populations, respectively.

- 1. Costs
     1. Methods

Unit costs were collected from a number of different sources including hospital price lists [46] and TLV Periodens vara [47], and Statistics Sweden. When hospital price lists are used, “utomlänspriser”, i.e. the cost for treatment outside a patient’s residential county, is used. All costs are inflated to 2013 and are given in Swedish kronor (SEK).

- - 1. General ward

Unit costs for inpatient hospital services were obtained from hospital price lists. In all cases, the cost is given as the cost per bed day.

Unit costs for treatment in a general ward are given as admission costs and costs per bed day. For the first day, the total cost will be the sum of the admission cost and the cost per bed day. For subsequent days, only the cost per bed day applies. An average cost per bed day was assumed for simplicity.

Table 12 summarises ward costs by care setting. The following admission locations are assumed:

- ‘All SIADH’: internal medicine
- SCLC: Oncology clinic
- Pneumonia: pulmonary care clinic

In practice, these costs were fairly similar (Table 12), and therefore choice of ward was not a large determinant of the cost-effectiveness results.

Table 12: General Hospital ward cost per bed day

| Resource | Admission cost (SEK) | Cost per bed day (SEK) | Total cost, day 1 (SEK) | Total cost, day 2+ (SEK) | Source |
| --- | --- | --- | --- | --- | --- |
| Internal medicine clinic | 1,706 | 5,483 | 7,190 | 5,483 | Southern region price list [46] |
| Pulmonary care clinic | 2,581 | 3,351 | 5,932 | 3,351 | Southern region price list [46] |
| Oncology clinic | 1,652 | 5,028 | 6,681 | 5,028 | Southern region price list [46] |

Abbreviations: SEK, Swedish kronor

- - 1. Palliative care

Unit costs for palliative care were obtained from the Southern region price list. Costs are given as cost per bed day.

Table 13: Palliative care cost per bed day

| Resource | Unit cost (SEK) | Source |
| --- | --- | --- |
| Hospital-based palliative activities cost/day | 7,381 | Southern region price list [46] |
| ASIH (advanced care at home) | 3,505 | Southern region price list [46] |

Abbreviations: SEK, Swedish kronor

- - 1. Chemotherapy in SCLC

Chemotherapy drug costs were taken from the periodens vara [47]. Chemotherapy drug costs were obtained by finding the product and pack size with the lowest unit price among the listed primary choices for October 2013 (Table 14). Chemotherapy costs were applied at every inpatient visit. This assumption ignores the fact that many patients will receive chemotherapy in the outpatient setting. However, because no difference in readmissions is included, the incremental effect of this assumption is minimal. Similarly, it is simply assumed that chemotherapy is applied for a maximum of 84 days (3 months). However, because the model assumes no survival benefit associated with [Na^+^] correction, the incremental effect of this assumption is minimal.

Table 14: Drug costs

| Substance | Product name | Pack size (mg) | Cost/pack (SEK) | Cost/mg (SEK) | Source |
| --- | --- | --- | --- | --- | --- |
| Cisplatin | Cisplatin Hospira | 100 | 422 | 4.22 | TLV ‘Periodens vara’ October 2013 [47] |
| Carboplatin | Carboplatin Teva | 600 | 1538 | 2.56 | TLV ‘Periodens vara’ October 2013 [47] |
| Etoposide | Eposin | 500 | 710.50 | 1.42 | TLV ‘Periodens vara’ October 2013 [47] |

Abbreviations: SEK, Swedish kronor

In assessing the cost per cycle for SCLC chemotherapy treatment, the calculations were based on the treatment recommendations for Southern Sweden [48]. According to the treatment protocol, SCLC stage I-III disease is treated with chemotherapy and radiotherapy given either in sequence or concomitantly.

- The choice of chemotherapy is either cisplatin and etoposide combined or carboplatin and etoposide combined
- Each treatment cycle runs for 21 days, in which cisplatin/carboplatin is given at day 1 while etoposide is given during days 1-3
- The daily dose of cisplatin is 75 mg/m^2^ body surface (sequential chemo- and radiotherapy) or 60 mg/m^2^ body surface (concomitant chemo- and radiotherapy)
- The daily dose of carboplatin is 5 AUC min×mg/mL (sequential chemo- and radiotherapy)
- The daily dose of etoposide is 100 mg/m^2^ body surface (sequential and concomitant chemo- and radiotherapy)

A UK study of 3,600 adult patients with cancer from 2005 reported an average body surface area of 1.79 m^2^ [49], which was used in the dose calculations for cisplatin and etoposide. The dose of carboplatin was calculated according to Calvert’s formula, using a GFR of 100 mL/min. The hospital cost was obtained from the Southern region price list (three days of day clinic care with a unit cost of SEK 2,976) [46].

Table 15: Cost of a cycle of chemotherapy for SCLC

| Protocol | Cisplatin cost/cycle (SEK) | Carboplatin cost/cycle (SEK) | Etoposide cost/cycle (SEK) | Day clinic care, cost/cycle (SEK) | Total cost/cycle (SEK) |
| --- | --- | --- | --- | --- | --- |
| Cisplatin/Etoposide, sequential chemo-/radiotherapy | 567 | - | 763 | 8,928 | 10,276 |
| Cisplatin+Etoposide, concomitant chemo-/radiotherapy | 453 | - | 763 | 8,928 | 10,144 |
| Carboplatin+Etoposide, sequential chemo-/radiotherapy | - | 1,602 | 763 | 8,928 | 11,293 |

Abbreviations: SEK, Swedish kronor

- - 1. Monitoring

The laboratory cost for the blood test was obtained from the Southern region price list (Table 16) [46]. Monitoring is assumed to occur more frequently in tolvaptan patients during the first day of treatment, based on clinical expert opinion. It is assumed that [Na^+^] monitoring would occur four times on day 1 of treatment with tolvaptan (every 6 hours, as specified within the tolvaptan Summary of Product Characteristics [50]), reducing to once per day thereafter. For NAT, it is assumed that [Na^+^] monitoring would occur twice on day one, and once daily thereafter.

Table 16: Blood test costs

| Resource | Unit cost (SEK) | Source |
| --- | --- | --- |
| Blood test for [Na^+^] | 13 | Southern region price list [46], clinical chemistry |

Abbreviations: SEK, Swedish kronor

- - 1. Productivity

The cost of productivity losses due to absenteeism can be calculated from the average monthly salary (SEK 40,763 including social fees and pension) in Sweden; SEK 2,163 per work day. This calculation is based on a mean of 226.18 working days per year.

It is assumed 17% of ‘all SIADH’ and pneumonia patients are in work between admissions, based on the labour participation on individuals in the 65-74 years age range [51]. It was assumed that SCLC patients would not be in employment.

- - 1. Costs of tolvaptan

The base case analysis assumes that tolvaptan is used at licensed doses of 15 or 30 mg daily. Tolvaptan licensed doses are 15-60 mg; however doses of 60 mg are not believed to be used widely in clinical practice: in the PASS, <3% of patients were prescribed a daily dose exceeding 30 mg and <1% of patients were prescribed a 60 mg daily dose [unpublished observations, Otsuka]. No titration period is assumed. The costs of 15 and 30 mg formulations of tolvaptan are identical so titration ‘up’ to 30 mg from 15 mg would not be associated with increased drug costs. In order to estimate the price of tolvaptan in Sweden we assume that pharmacies apply the same margin as for drugs within the reimbursement system. The pharmacy purchasing price of tolvaptan in Sweden is SEK 9,980 [unpublished observations, Otsuka]; applying the TLV mandated pharmacy margin^[[8]](#endnote-8)^ the cost of one pack of 10 tablets of tolvaptan (15 or 30 mg) is SEK 10,147. The daily cost of tolvaptan treatment is therefore assumed to be SEK 1,015. No additional cost is applied in the NAT arm of the model. Table 17 presents drug acquisition costs.

Table 17: Tolvaptan acquisition costs

| Formulation | Dose/tab (mg) | Tabs/pack | Pack cost | Price / tab |
| --- | --- | --- | --- | --- |
| tolvaptan 15mg | 15.00 | 10.00 | SEK 10,147 | SEK 1,014.70 |
| tolvaptan 30mg | 30.00 | 10.00 | SEK 10,147 | SEK 1,014.70 |

- - 1. Scenario analyses

A number of alternative scenarios were tested. These are summarised in Table 18.

Table 18: Summary of alternative scenarios tested

| Short name | Description |
| --- | --- |
| Base case | All parameters at default values |
| Time horizon=60 days | Time horizon=60 days |
| Time horizon=90 days | Time horizon=90 days |
| Time horizon=180 days | Time horizon=180 days |
| Time horizon=365 days | Time horizon=365 days |
| Duration tolvaptan treatment: PASS | Duration tolvaptan treatment taken from PASS (generalised gamma distribution) |
| Hospital LOS from pooled SIADH SALT I & II population | Use of overall population LOS data from Verbalis et al (6.19 NAT; 4.98 tolvaptan), which include baseline [Na^+^] >130 mmol/L |
| Baseline patient characteristics: HN Registry | All baseline patient characteristics taken from HN Registry (instead of ART) |
| No hospital LOS treatment benefit | No hospital LOS benefit for tolvaptan treatment assumed |
| tolvaptan HRQL -25% | Reduction in size of tolvaptan HRQL coefficient by 25% |
| No early tolvaptan treatment discontinuation rule | No early tolvaptan treatment discontinuation rule applied |
| Hospital LOS for NAT +50% | Hospital LOS for NAT + 50% |
| Hospital LOS for NAT -50% | Hospital LOS for NAT - 50% |
| Tolvaptan HRQL +25% | Increase in size of tolvaptan HRQL coefficient by 25% |
| Tolvaptan LOS benefit: 10% reduction vs. NAT | Percentage reduction in hospital LOS with tolvaptan treatment=10% |
| Tolvaptan LOS benefit: 15% reduction vs. NAT | Percentage reduction in hospital LOS with tolvaptan treatment=15% |
| Tolvaptan LOS benefit: 25% reduction vs. NAT | Percentage reduction in hospital LOS with tolvaptan treatment=25% |
| Tolvaptan LOS benefit: 30% vs. NAT | Percentage reduction in hospital LOS with tolvaptan treatment=30% |
| Duration treatment tolvaptan +50% | Increase in duration of tolvaptan treatment by 50% |
| Duration treatment tolvaptan -50% | Reduction in duration of tolvaptan treatment by 50% |
| 30-day duration of tolvaptan treatment (long-term SCLC) | Duration of tolvaptan treatment is 30 days and probability of resolution is 30% in SCLC population to replicate long-term treatment scenario |
| All costs +25% | All costs +25% (except cost of tolvaptan) |
| All costs -25% | All costs -25% (except cost of tolvaptan) |

Abbreviations: LOS, length of stay; SIADH, syndrome of inappropriate antidiuretic hormone secretion; SCLC, small-cell lung cancer.

1. Results
   1. Base case results

Base case results for the ‘all SIADH’ population are presented in Table 19 based on 100,000 hypothetical patients. Tolvaptan was associated with reduced costs and increased incremental QALYs versus NAT. Tolvaptan was therefore considered to be the dominant strategy. Table 20 and

Table 21 present results for the SCLC and pneumonia subpopulations, respectively. In both of these subpopulations, tolvaptan was associated with costs saving and QALY improvements, and tolvaptan was therefore the dominant strategy.

Table 19: Base case results, ‘all SIADH’ population

| Item | NAT | Tolvaptan | Incremental |
| --- | --- | --- | --- |
| Prescription costs | SEK 0 | SEK 3,234 | SEK 3,234 |
| [Na^+^] monitoring | SEK 137 | SEK 150 | SEK 13 |
| Inpatient costs | SEK 52,742 | SEK 44,276 | -SEK 8,466 |
| Palliative care | SEK 0 | SEK 0 | SEK 0 |
| Chemotherapy | SEK 0 | SEK 0 | SEK 0 |
| Lost productivity savings | -SEK 7,417 | -SEK 7,977 | -SEK 560 |
| **Total costs** | **SEK 45,462** | **SEK 39,683** | **-SEK 5,779** |
| Life-years (discounted) | 0.081 | 0.080 | -0.001 |
| Cumulative inpatient days whilst in model | 9.2 | 7.7 | -1.5 |
| Mean time to readmission (days) | 213.8 | 214.2 | 0.4 |
| Mean LOS^†^ (days) | 8.0 | 6.4 | -1.6 |
| % patients with corrected HN | 18.0% | 68.9% | 50.9% |
| Mean duration tolvaptan treatment (days) | NA | 2.6 | NA |
| Mean days in model | 29.4 | 29.4 | 0.0 |
| **QALYs** | **0.05381** | **0.05567** | **0.00186** |
| **ICER** |  |  | **Dominant** |

Abbreviations: HN, hyponatraemia; ICER, incremental cost-effectiveness ratio; LOS, length of stay; NAT, no active treatment; QALY, quality-adjusted life life-year; SEK, Swedish kronor; SIADH, syndrome of inappropriate antidiuretic hormone secretion.

† Index admission

Table 20: Base case results, SCLC cohort

| Item | NAT | Tolvaptan | Incremental |
| --- | --- | --- | --- |
| Prescription costs | SEK 0 | SEK 6,947 | SEK 6,947 |
| [Na^+^] monitoring | SEK 245 | SEK 264 | SEK 19 |
| Inpatient costs | SEK 86,724 | SEK 71,662 | -SEK 15,062 |
| Palliative care | SEK 263,111 | SEK 262,450 | -SEK 661 |
| Chemotherapy | SEK 22,637 | SEK 22,983 | SEK 346 |
| Lost productivity savings | SEK 0 | SEK 0 | SEK 0 |
| **Total costs** | **SEK 372,717** | **SEK 364,306** | **-SEK 8,411** |
| Life-years (discounted) | 0.315 | 0.314 | -0.001 |
| Cumulative inpatient days whilst in model | 16.6 | 13.6 | -3.0 |
| Mean time to readmission (days) | 101.0 | 100.0 | -1.0 |
| Mean LOS^†^ (days) | 8.0 | 6.4 | -1.6 |
| % Corrected HN | 18.4% | 69.2% | 50.8% |
| Mean duration tolvaptan treatment (days) | NA | 3.3 | NA |
| Mean days in model | 115.6 | 115.3 | -0.3 |
| **QALYs** | **0.21817** | **0.22101** | **0.00284** |
| **ICER** |  |  | **Dominant** |

Abbreviations: HN, hyponatraemia; ICER, incremental cost-effectiveness ratio; LOS, length of stay; NAT, no active treatment; QALY, quality-adjusted life life-year; SCLC, small cell lung cancer; SEK, Swedish kronor.

† Index admission

Table 21: Base case results, pneumonia cohort

| Item | NAT | Tolvaptan | Incremental |
| --- | --- | --- | --- |
| Prescription costs | SEK 0 | SEK 3,109 | SEK 3,109 |
| [Na^+^] monitoring | SEK 135 | SEK 147 | SEK 12 |
| Inpatient costs | SEK 33,678 | SEK 28,435 | -SEK 5,243 |
| Palliative care | SEK 0 | SEK 0 | SEK 0 |
| Chemotherapy | SEK 0 | SEK 0 | SEK 0 |
| Lost productivity savings | -SEK 7,272 | -SEK 7,828 | -SEK 556 |
| **Total costs** | **SEK 26,541** | **SEK 23,863** | **-SEK 2,678** |
| Life-years (discounted) | 0.079 | 0.079 | 0.000 |
| Cumulative inpatient days whilst in model | 9.1 | 7.5 | -1.6 |
| Mean time to readmission (days) | 144.9 | 144.4 | -0.5 |
| Mean LOS^†^ (days) | 8.0 | 6.4 | -1.6 |
| % Corrected HN | 19.0% | 70.3% | 51.3% |
| Mean duration tolvaptan treatment (days) | NA | 2.5 | NA |
| Mean days in model | 28.9 | 28.9 | -0.0 |
| **QALYs** | **0.05689** | **0.05868** | **0.00179** |
| **ICER** |  |  | **Dominant** |

Abbreviations: HN, hyponatraemia; ICER, incremental cost-effectiveness ratio; LOS, length of stay; NAT, no active treatment; QALY, quality-adjusted life life-year; SEK, Swedish kronor.

† Index admission

- 1. Scenario analyses

Table 22 presents results of scenario analysis by population. Each scenario is based on results from 30,000 hypothetical patients. The most important determinants of cost-effectiveness appear to be:

- Reduction in hospital LOS associated with tolvaptan
  - Assuming no LOS benefit for tolvaptan resulted in large ICERs (>SEK 1,400,000) in all populations
- Duration of treatment with tolvaptan
  - Use of PASS data led to an ICER of SEK 1,424,604 in the ‘all SIADH’ population
  - Increasing the duration of treatment with tolvaptan by 50% did not change the results in the SCLC and pneumonia populations (tolvaptan was the dominant strategy in both populations)
- Long-term SCLC treatment scenario

The 30-day long-term SCLC treatment scenario led to an ICER of SEK 2,852,418

Table 22: Results of scenario analyses by population

|  | All SIADH | | | SCLC | | | Pneumonia | | |
| --- | --- | --- | --- | --- | --- | --- | --- | --- | --- |
| Scenario Analysis | Δ costs | ΔQALYs | ICER | Δ costs | ΔQALYs | ICER | Δ costs | ΔQALYs | ICER |
| Base case^†^ | -SEK 5,778 | 0.0019 | Dominant | -SEK 8,412 | 0.0028 | Dominant | -SEK 2,677 | 0.0018 | Dominant |
| Time horizon=60 days | -SEK 7,319 | 0.0021 | Dominant | -SEK 5,702 | 0.0020 | Dominant | -SEK 3,760 | 0.0023 | Dominant |
| Time horizon=90 days | -SEK 9,478 | 0.0027 | Dominant | -SEK 6,534 | 0.0023 | Dominant | -SEK 4,092 | 0.0024 | Dominant |
| Time horizon=180 days | -SEK 11,181 | 0.0029 | Dominant | -SEK 9,633 | 0.0036 | Dominant | -SEK 5,645 | 0.0032 | Dominant |
| Time horizon=365 days | -SEK 15,238 | 0.0042 | Dominant | -SEK 13,070 | 0.0033 | Dominant | -SEK 8,269 | 0.0037 | Dominant |
| Duration tolvaptan treatment: PASS | SEK 2,754 | 0.0019 | SEK 1,424,604 | NA | NA | NA | NA | NA | NA |
| Hospital LOS from pooled SIADH SALT I & II population | -SEK 4,154 | 0.0015 | Dominant | -SEK 5,043 | 0.0017 | Dominant | -SEK 1,855 | 0.0015 | Dominant |
| Baseline patient characteristics: HN Registry | -SEK 6,114 | 0.0020 | Dominant | -SEK 9,565 | 0.0036 | Dominant | -SEK 2,677 | 0.0018 | Dominant |
| No hospital LOS treatment benefit | SEK 2,941 | 0.002 | SEK 1,490,024 | SEK 5,741 | 0.004 | SEK 1,441,596 | SEK 2,999 | 0.0019 | SEK 1,549,236 |
| tolvaptan HRQL -25% | -SEK 6,181 | 0.0015 | Dominant | -SEK 9,633 | 0.0027 | Dominant | -SEK 2,729 | 0.0014 | Dominant |
| No early tolvaptan treatment discontinuation rule | -SEK 5,613 | 0.0020 | Dominant | -SEK 8,074 | 0.0036 | Dominant | -SEK 2,186 | 0.0018 | Dominant |
| Hospital LOS for NAT + 50% | -SEK 7,463 | 0.0027 | Dominant | -SEK 16,086 | 0.0056 | Dominant | -SEK 3,949 | 0.0027 | Dominant |
| Hospital LOS for NAT - 50% | -SEK 2,409 | 0.0009 | Dominant | -SEK 2,633 | 0.0011 | Dominant | -SEK 245 | 0.0009 | Dominant |
| tolvaptan HRQL +25% | -SEK 6,181 | 0.0025 | Dominant | -SEK 9,633 | 0.0045 | Dominant | -SEK 2,729 | 0.0023 | Dominant |
| Hospital LOS benefit: 10% | -SEK 1,667 | 0.0020 | Dominant | -SEK 1,994 | 0.0037 | Dominant | SEK 375 | 0.0019 | SEK 198,726 |
| Hospital LOS benefit: 15% | -SEK 3,831 | 0.0020 | Dominant | -SEK 5,812 | 0.0035 | Dominant | -SEK 1,076 | 0.0019 | Dominant |
| Hospital LOS benefit: 25% | -SEK 8,546 | 0.0020 | Dominant | -SEK 13,494 | 0.0032 | Dominant | -SEK 4,196 | 0.0018 | Dominant |
| Hospital LOS benefit: 30% | -SEK 10,996 | 0.0020 | Dominant | -SEK 18,123 | 0.0032 | Dominant | -SEK 5,803 | 0.0017 | Dominant |
| Duration tolvaptan +50% | NA | NA | NA | -SEK 6,756 | 0.0036 | Dominant | -SEK 1,443 | 0.0018 | Dominant |
| Duration tolvaptan -50% | NA | NA | NA | -SEK 12,710 | 0.0036 | Dominant | -SEK 4,125 | 0.0018 | Dominant |
| 30-day duration of tolvaptan treatment (long-term SCLC) | NA | NA | NA | SEK 21,302 | 0.0075 | SEK 2,852,418 | NA | NA | NA |
| All costs +25% | -SEK 8,382 | 0.0020 | Dominant | -SEK 8,702 | 0.0036 | Dominant | -SEK 4,047 | 0.0018 | Dominant |
| All costs -25% | -SEK 3,980 | 0.0020 | Dominant | -SEK 9,960 | 0.0036 | Dominant | -SEK 1,411 | 0.0018 | Dominant |

Abbreviations: ICER, incremental cost-effectiveness ratio; LOS, length of stay; QALYs, quality-adjusted life-years; SCLC, small-cell lung cancer; SIADH, syndrome of inappropriate antidiuretic hormone secretion.
†Note that these results are the same as the base case results because this analysis uses the same number of hypothetical patients (n=100,000), but please note subsequent scenarios were performed with n=30,000 hypothetical patient

1. References

1. Makin A, Verbalis J, Greenberg A, Grohe C: **Small Cell Lung Cancer and Hyponatremia: Interim Results From a Prospective, Observational, Global Registry**. *Presented at the European Multidisciplinary Conference in Thoracic Oncology 2013; May 9–11, 2013; Lugano, Switzerland* 2013.

2. Schrier RW, Gross P, Gheorghiade M, Berl T, Verbalis JG, Czerwiec FS, Orlandi C, Salt Investigators: **Tolvaptan, a selective oral vasopressin V2-receptor antagonist, for hyponatremia**. *The New England journal of medicine* 2006, **355**(20):2099-2112.

3. Doshi SM, Shah P, Lei X, Lahoti A, Salahudeen AK: **Hyponatremia in hospitalized cancer patients and its impact on clinical outcomes**. *American Journal of Kidney Diseases* 2012, **59**(2):222-228.

4. Gheorghiade M GS, Udelson JE, Konstam MA, Czerwiec F, Ouyang J, Orlandi C, Tolvaptan Investigators: **Vasopressin v(2) receptor blockade with tolvaptan versus fluid restriction in the treatment of hyponatremia**. *The American journal of cardiology* 2006, **97**(7):1064-1067.

5. Hansen O, Sorensen P, Hansen KH: **The occurrence of hyponatremia in SCLC and the influence on prognosis: a retrospective study of 453 patients treated in a single institution in a 10-year period**. *Lung Cancer* 2010, **68**(1):111-114.

6. Hermes A, Waschki B, Reck M: **Hyponatremia as prognostic factor in small cell lung cancer - A retrospective single institution analysis**. *Respiratory Medicine* 2012, **106**(6):900-904.

7. Verbalis JG, Adler S, Schrier RW, Berl T, Zhao Q, Czerwiec FS, Investigators S: **Efficacy and safety of oral tolvaptan therapy in patients with the syndrome of inappropriate antidiuretic hormone secretion**. *European journal of endocrinology / European Federation of Endocrine Societies* 2011, **164**(5):725-732.

8. Latimer N: **NICE DSU Technical support document 14: survival analysis for economic evaluation alongside clinical trials - extrapolation with patient-level data. Report by the Decision Support Unit**. 2011.

9. Akaike H: **A new look at the statistical model identification**. *IEEE Transactions on Automatic Control* 1974, **19**(6):716-723.

10. Petereit C, Zaba O, Teber I, Luders H, Grohe C: **A rapid and efficient way to manage hyponatremia in patients with SIADH and small cell lung cancer: treatment with tolvaptan**. *BMC Pulm Med* 2013, **13**:55.

11. Borenstein J, Aronow HU, Bolton LB, Choi J, Bresee C, Braunstein GD: **Early recognition of risk factors for adverse outcomes during hospitalization among Medicare patients: a prospective cohort study**. *BMC geriatr* 2013, **13**:72.

12. Estrada C, Adewale A, Petry M, Abebe M, Wadhwa NK: **Hyponatremia management: Is it still an enigma?** *American Journal of Kidney Diseases* 2013, **61 (4)**:A39.

13. Hackworth WA, Heuman DM, Sanyal AJ, Fisher RA, Sterling RK, Luketic VA, Shiffman ML, Maluf DG, Cotterell AH, Posner MP *et al*: **Effect of hyponatraemia on outcomes following orthotopic liver transplantation**. *Liver International* 2009, **29**(7):1071-1077.

14. Kao L, Al-Lawati Z, Vavao J, Steinberg GK, Katznelson L: **Prevalence and clinical demographics of cerebral salt wasting in patients with aneurysmal subarachnoid hemorrhage**. *Pituitary* 2009, **12**(4):347-351.

15. Nair V, Niederman MS, Masani N, Fishbane S: **Hyponatremia in community-acquired pneumonia**. *American Journal of Nephrology* 2007, **27**(2):184-190.

16. Salahudeen AK, Doshi SM, Shah P: **The frequency, cost, and clinical outcomes of hypernatremia in patients hospitalized to a comprehensive cancer center**. *Supportive Care in Cancer* 2013, **21**(7):1871-1878.

17. Sherlock M, O'Sullivan E, Agha A, Behan LA, Rawluk D, Brennan P, Tormey W, Thompson CJ: **The incidence and pathophysiology of hyponatraemia after subarachnoid haemorrhage**. *Clinical Endocrinology* 2006, **64**(3):250-254.

18. Stelfox HT, Ahmed SB, Khandwala F, Zygun D, Shahpori R, Laupland K: **The epidemiology of intensive care unit-acquired hyponatraemia and hypernatraemia in medical-surgical intensive care units**. *Critical Care* 2008, **12**(6).

19. Stelfox HT, Ahmed SB, Zygun D, Khandwala F, Laupland K: **Characterization of intensive care unit acquired hyponatremia and hypernatremia following cardiac surgery**. *Canadian Journal of Anesthesia* 2010, **57**(7):650-658.

20. Wald R, Jaber BL, Price LL, Upadhyay A, Madias NE: **Impact of hospital-associated hyponatremia on selected outcomes**. *Archives of Internal Medicine* 2010, **170**(3):294-302.

21. Zilberberg MD, Exuzides A, Spalding J, Foreman A, Jones AG, Colby C, Shorr AF: **Hyponatremia and hospital outcomes among patients with pneumonia: a retrospective cohort study**. *BMC pulmonary medicine* 2008, **8**:16.

22. Hagino T, Ochiai S, Watanabe Y, Senga S, Saito M, Takayama Y, Wako M, Ando T, Sato E, Haro H: **Hyponatremia at admission is associated with in-hospital death in patients with hip fracture**. *Archives of Orthopaedic and Trauma Surgery* 2013, **133**(4):507-511.

23. Chua M, Hoyle GE, Soiza RL: **Prognostic implications of hyponatremia in elderly hospitalized patients**. *Archives of Gerontology and Geriatrics* 2007, **45**(3):253-258.

24. Olsson K, Ohlin B, Melander O: **Epidemiology and characteristics of hyponatremia in the emergency department**. *European Journal of Internal Medicine* 2013, **24**(2):110-116.

25. Turgutalp K, Ozhan O, Oguz EG, Horoz M, Camsari A, Yilmaz A, Kiykim A, Arici M: **Clinical features, outcome and cost of hyponatremiaassociated admission and hospitalization in elderly and very elderly patients: A single-center experience in Turkey**. *International Urology and Nephrology* 2013, **45**(1):265-273.

26. List AF, Hainsworth JD, Davis BW, Hande KR, Greco FA, Johnson DH: **The syndrome of inappropriate secretion of antidiuretic hormone (SIADH) in small-cell lung cancer**. *Journal of clinical oncology : official journal of the American Society of Clinical Oncology* 1986, **4**(8):1191-1198.

27. Tai P, Yu E, Jones K, Sadikov E, Mahmood S, Tonita J: **Syndrome of inappropriate antidiuretic hormone secretion (SIADH) in patients with limited stage small cell lung cancer**. *Lung Cancer* 2006, **53**(2):211-215.

28. Gheorghiade M, Rossi JS, Cotts W, Shin DD, Hellkamp AS, Pina IL, Fonarow GC, DeMarco T, Pauly DF, Rogers J *et al*: **Characterization and prognostic value of persistent hyponatremia in patients with severe heart failure in the ESCAPE trial**. *Archives of Internal Medicine* 2007, **167**(18):1998-2005.

29. Scherz N, Labarere J, Mean M, Ibrahim SA, Fine MJ, Aujesky D: **Prognostic importance of hyponatremia in patients with acute pulmonary embolism**. *American Journal of Respiratory and Critical Care Medicine* 2010, **182**(9):1178-1183.

30. Funk GC, Lindner G, Druml W, Metnitz B, Schwarz C, Bauer P, Metnitz PG: **Incidence and prognosis of dysnatremias present on ICU admission**. *Intensive care medicine* 2010, **36**(2):304-311.

31. Hampshire PA, Welch CA, McCrossan LA, Francis K, Harrison DA: **Admission factors associated with hospital mortality in patients with haematological malignancy admitted to UK adult, general critical care units: A secondary analysis of the ICNARC Case Mix Programme Database**. *Critical Care* 2009, **13**(4).

32. Zilberberg MD, Exuzides A, Spalding J, Foreman A, Jones AG, Colby C, Shorr AF: **Epidemiology, clinical and economic outcomes of admission hyponatremia among hospitalized patients**. *Curr Med Res Opin* 2008, **24**(6):1601-1608.

33. Hoorn EJ, Lindemans J, Zietse R: **Development of severe hyponatraemia in hospitalized patients: treatment-related risk factors and inadequate management**. *Nephrology, dialysis, transplantation : official publication of the European Dialysis and Transplant Association - European Renal Association* 2006, **21**(1):70-76.

34. Akaza H, Tsukamoto T, Fujioka T, Tomita Y, Kitamura T, Ozono S, Miki T, Naito S, Zembutsu H, Nakamura Y: **Combined immunotherapy with low-dose IL-2 plus IFN-alpha for metastatic renal cell carcinoma: survival benefit for selected patients with lung metastasis and serum sodium level**. *Japanese Journal of Clinical Oncology* 2011, **41**(8):1023-1030.

35. Gill G, Huda B, Boyd A, Skagen K, Wile D, Watson I, van Heyningen C: **Characteristics and mortality of severe hyponatraemia--a hospital-based study**. *Clinical Endocrinology* 2006, **65**(2):246-249.

36. Havranek S, Belohlavek J, Skulec R, Kovarnik T, Dytrych V, Linhart A: **Long-term prognostic impact of hyponatremia in the ST-elevation myocardial infarction**. *Scand J Clin Lab Invest* 2011, **71**(1):38-44.

37. Kawashima A, Takayama H, Arai Y, Nin M, Tanigawa G, Yasunaga Y, Mukai M, Nomura H, Oka D, Yoshioka T *et al*: **Impact of hyponatremia on survival of patients with metastatic renal cell carcinoma treated with molecular targeted therapy**. *Journal of Urology* 2012, **1)**:e806.

38. Doshi SM, Shah P, Lei X, Lahoti A, Salahudeen AK: **Hyponatremia in hospitalized cancer patients and its impact on clinical outcomes**. *American Journal of Kidney Diseases* 2012, **59**(2):222-228.

39. Statistics Sweden: **Life tables for 2012, divided into men and women. http://www.scb.se/en_/Finding-statistics/Statistics-by-subject-area/Population/Population-composition/Population-statistics/Aktuell-Pong/25795/**. 2012.

40. Trueman D, Livings C, Mildred M: **PRM81 Methods of Obtaining Evidence From Published Survival Data for Use in Decision Analytic Models**. *Value in Health* 2012, **15**(7):A475-A475.

41. Gray AM, Rivero-Arias O, Clarke PM: **Estimating the Association between SF-12 Responses and EQ-5D Utility Values by Response Mapping**. *Medical Decision Making* 2006, **26**:18-29.

42. Dolan P: **Modeling valuations for EuroQol health states**. *Medical care* 1997, **35**(11):1095-1108.

43. Trueman D, Hancock E, Robinson P, Dale P, O'Reilly K, Gisby M: **EQ-5D scores in patients receiving tolvaptan for the treatment of hyponatraemia secondary to the Syndrome of Inappropriate Antidiuretic Hormone Secretion. Poster presented at ISPOR, Oct 2014, Amsterdam**.

44. Loveman E, Jones J, Hartwell D, Bird A, Harris P, Welch K, Clegg A: **The clinical effectiveness and cost-effectiveness of topotecan for small cell lung cancer: a systematic review and economic evaluation**. *Health Technol Assess* 2010, **14**(19):1-204.

45. Schuetz P, Albrich W, Suter I, Hug B, Christ-Crain M, Holler T, Henzen C, Krause M, Schoenenberger R, Zimmerli W *et al*: **Quality of care delivered by fee-for-service and DRG hospitals in Switzerland in patients with community-acquired pneumonia**. *Swiss Medical Weekly* 2011.

46. Southern region health board: **Southern region price list**. *Available at http://wwwskanese/Upload/Webbplatser/Sodra%20regionvardsnamnden/prislista/2013/helaprislistan2013pdf* 2013.

47. Tandvårds- och läkemedelsförmånsverket (TLV): **Periodens vara**. *Available at http://wwwtlvse/apotek/utbyte-av-lakemedel-pa-apotek/periodens-varor/*.

48. **Regionalt vårdprogram för lungcancer, Region Skåne**. 2011.

49. Sacco JJ, Botten J, Macbeth F, Bagust A, Clark P: **The average body surface area of adult cancer patients in the UK: a multicentre retrospective study**. *PLoS One* 2010, **5**(1):e8933.

50. Otsuka Pharmaceuticals (UK) Ltd: **Summary of Product Characteristics: Samsaca 15 mg and 30 mg tablets**. 2013.

51. Statistics Sweden: **Labour Force Survey October 2013**. *Available at http://wwwscbse/en_/Finding-statistics/Statistics-by-subject-area/Labour-market/Labour-force-surveys/Labour-Force-Survey-LFS/* 2013.

1. 3 Endnotes

   Identified studies all used slightly different definitions of [Na+] correction. Doshi et al [3] define [Na+] correction as reaching [Na+] ≥135 mEq/L during admission. Gheorghiade et al [4] define correction as achieving [Na^+^] >134 mEq/L during the hospitalisation (following admission with [Na^+^] ≤134 mEq/L). Hansen et al [5] define correction as achieving plasma [Na^+^] ≥136 mEq/L at the time of second cycle of chemotherapy. Hermes et al [6] define normal [Na^+^] as ≥135 mmol/L. [↑](#endnote-ref-1)
2. Probability of [Na^+^] correction (11.5% and 60.0% in NAT and tolvaptan arms, respectively) was taken from Verbalis et al and converted to odds (using odds=probability/(1-probability)) before the ratio of these is taken to provide the odds ratio [↑](#endnote-ref-2)
3. The estimated power was 0.0899, estimated using Satterthwaite's t test assuming unequal variances [↑](#endnote-ref-3)
4. In the SCLC scenario considering longer-term treatment, it is assumed that 30% of patients would experience resolution of HN secondary to SIADH at discharge [↑](#endnote-ref-4)
5. This study therefore included mixed aetiologies [↑](#endnote-ref-5)
6. In order to estimate HRQL for SCLC and HN secondary to SIADH, the effect size associated with tolvaptan® was subtracted from the baseline HRQL with SLCLC: 0.7 - 0.0948 [↑](#endnote-ref-6)
7. In order to estimate HRQL for pneumonia and HN secondary to SIADH, the effect size associated with tolvaptan® was subtracted from the baseline HRQL with pneumonia: 0.82 - 0.0948 [↑](#endnote-ref-7)
8. Available from http://www.tlv.se/apotek/rakna-ut-aup/#result [↑](#endnote-ref-8)
